# *In vivo* liposomal delivery of PPARα/γ dual agonist tesaglitazar in a model of obesity enriches macrophage targeting and limits liver and kidney drug effects

**DOI:** 10.7150/thno.36572

**Published:** 2020-01-01

**Authors:** Victoria Osinski, Dustin K. Bauknight, Siva Sai Krishna Dasa, Matthew J. Harms, Tobias Kroon, Melissa A. Marshall, James C. Garmey, Anh T. Nguyen, Julia Hartman, Aditi Upadhye, Prasad Srikakulapu, Andrea Zhou, Gavin O'Mahony, Alexander L. Klibanov, Kimberly A. Kelly, Jeremie Boucher, Coleen A. McNamara

**Affiliations:** 1 Robert M. Berne Cardiovascular Research Center, University of Virginia, Charlottesville, Virginia, 22908, USA; 2 Department of Pathology, University of Virginia, Charlottesville, VA, 22908, USA; 3 Department of Biomedical Engineering, University of Virginia, Charlottesville, Virginia, 22908, USA; 4 Research and Early Development, Cardiovascular, Renal and Metabolism (CVRM), BioPharmaceuticals R&D, AstraZeneca, Gothenburg, Sweden; 5 Department of Medicine, Division of Cardiovascular Medicine, University of Virginia, Charlottesville, VA, USA; 6 The Lundberg Laboratory for Diabetes Research, University of Gothenburg, Sweden; 7 Wallenberg Centre for Molecular and Translational Medicine, University of Gothenburg, Sweden

**Keywords:** liposomes, tesaglitazar, peroxisome proliferator-activated receptors, obesity-associated dysmetabolism, macrophages

## Abstract

Macrophages are important regulators of obesity-associated inflammation and PPARα and -γ agonism in macrophages has anti-inflammatory effects. In this study, we tested the efficacy with which liposomal delivery could target the PPARα/γ dual agonist tesaglitazar to macrophages while reducing drug action in common sites of drug toxicity: the liver and kidney, and whether tesaglitazar had anti-inflammatory effects in an *in vivo* model of obesity-associated dysmetabolism.

**Methods**: Male leptin-deficient (*ob/ob*) mice were administered tesaglitazar or vehicle for one week in a standard oral formulation or encapsulated in liposomes. Following the end of treatment, circulating metabolic parameters were measured and pro-inflammatory adipose tissue macrophage populations were quantified by flow cytometry. Cellular uptake of liposomes in tissues was assessed using immunofluorescence and a broad panel of cell subset markers by flow cytometry. Finally, PPARα/γ gene target expression levels in the liver, kidney, and sorted macrophages were quantified to determine levels of drug targeting to and drug action in these tissues and cells.

**Results**: Administration of a standard oral formulation of tesaglitazar effectively treated symptoms of obesity-associated dysmetabolism and reduced the number of pro-inflammatory adipose tissue macrophages. Macrophages are the major cell type that took up liposomes with many other immune and stromal cell types taking up liposomes to a lesser extent. Liposome delivery of tesaglitazar did not have effects on inflammatory macrophages nor did it improve metabolic parameters to the extent of a standard oral formulation. Liposomal delivery did, however, attenuate effects on liver weight and liver and kidney expression of PPARα and -γ gene targets compared to oral delivery.

**Conclusions**: These findings reveal for the first time that tesaglitazar has anti-inflammatory effects on adipose tissue macrophage populations *in vivo*. These data also suggest that while nanoparticle delivery reduced off-target effects, yet the lack of tesaglitazar actions in non-targeted cells such (as hepatocytes and adipocytes) and the uptake of drug-loaded liposomes in many other cell types, albeit to a lesser extent, may have impacted overall therapeutic efficacy. This fulsome analysis of cellular uptake of tesaglitazar-loaded liposomes provides important lessons for future studies of liposome drug delivery.

## Introduction

Liposomal drug delivery has emerged as a promising strategy to limit the side effects of otherwise effective therapeutics by directing the active compound to the cells and tissue of interest while avoiding others, such as the liver and kidney, which often contribute to undesired side effects 1, 2. With the addition of polyethylene glycol (PEG) to liposome formulations, half-life of circulating liposomes increases and uptake by the reticuloendothelial system (RES, comprised of the liver, kidneys, spleen, bone marrow, lungs, and lymph nodes) and free drug in circulation are reduced. Even with such advancements in liposome formulations, uptake of liposomes by the liver and other RES tissues and phagocytes is still prevalent 2. Liposomes enrich drug delivery to phagocytic cells such as tissue-resident macrophages including liver-resident macrophages known as Kupffer cells 3. Liposome delivery has been identified as a promising approach for diseases associated with macrophage dysfunction 4, 5. There is current interest in using targeted nanoparticle approaches to deliver compounds to both macrophages and a variety of non-macrophage cell types including cancer cells 6 and endothelial cells 7, but a thorough characterization of the cell types that take up liposomes *in vivo* has not been reported. In the context of obesity-associated dysmetabolism, a disease characterized and driven by macrophage dysfunction, the capacity to target drugs to macrophages or other non-phagocytic immune cell types including B cells and T cells in the adipose may prove useful as both cell types play important roles in regulating inflammation and macrophage recruitment to the adipose tissue during obesity 8. To address this, we used fluorescent labeling of liposomes coupled with fluorescently activated cell sorting (FACS), or flow cytometry, to thoroughly describe these cell types *in vivo* in an unbiased manner.

Furthermore, therapeutic compounds for obesity-associated dysmetabolism already exist, including the family of peroxisome proliferator-activated receptor (PPAR) agonists. These compounds act on nuclear receptors, PPARs, which respond to metabolites such as lipids and regulate expression of lipid and glucose metabolism genes 9. They are known to act on multiple tissues in humans and mice where they regulate lipid metabolism in the liver, triglyceride clearance, and insulin resistance to alleviate symptoms of diabetes and obesity-associated dysmetabolism 10, 11. There are three PPAR subtypes: PPARα, PPARγ, and PPARδ. While PPARα and PPARγ both effectively increase insulin sensitivity in tissues, PPARα activates fatty acid oxidation in the liver and PPARγ induces lipogenesis 9, 12. The family of PPARα/γ dual agonists, known as glitazars, includes a compound known as tesaglitazar, which lowers hyperglycemia and improves circulating lipid levels more effectively than PPARγ agonists such as pioglitazone 13, 14. However, increases in creatinine and reduction in glomerular filtration rate in subjects contributed to the termination of Phase III trials with this compound 13-20. Tesaglitazar has effects in the liver and kidneys of rodent models 21, 22, which make it a useful compound to study the effects of liposome formulation on the biodistribution and drug action among RES tissues. PPARα agonism profoundly induces expression of many lipid metabolism and transport genes in the murine liver and kidney including the *Fatty acid binding protein* family (*Fabp*), *lipoprotein lipase* (*Lpl*), *Enol-CoA hydratase and 3-hydroxyacyl CoA dehydrogenase* (*Ehhadh*), and *Pyruvate dehydrogenase kinase 4* (*Pdk4*) 23, 24. Additionally, PPARα agonists increase murine liver mass 10, an easily measured biomarker of liver target engagement. Nakashiro *et al*. demonstrated the efficacy of nanoparticle delivery of the PPARγ agonist pioglitazone to attenuate effects in the kidneys 25, but whether liposomal delivery of PPAR agonists attenuates drug action in the liver remains uninvestigated. Thus, to test whether liposomal delivery effectively reduces tesaglitazar-induced PPARα/γ agonism in the liver and kidney, we quantified liver mass and gene expression in the liver and kidney.

PPARα/γ agonism in the liver is known to be metabolically beneficial, however, previous literature reporting knockout of PPARγ in macrophages or *in vivo* treatment with a PPARγ agonist suggests that PPARα/γ agonism in macrophages (including Kupffer cells in the liver) might be sufficient to reduce obesity-associated dysmetabolism 26, 27. Macrophages are a key cellular regulator of obesity-associated inflammation 28 and reduction of adipose tissue macrophage (ATM) populations attenuates adipose tissue inflammation and insulin resistance 29, 30. A spectrum of ATM phenotypes ranging from pro-inflammatory CD11c^+^ M1 macrophages to anti-inflammatory, tissue resident CD301^+^ M2 macrophages exist in obese adipose tissue 31. M1 macrophages can be further divided by expression of CD206 (mannose receptor): CD11c^+^CD206^-^ M1a macrophages are characterized by increased expression of pro-inflammatory cytokines, while CD11c^+^CD206^+^ M1b macrophages are recruited to obese adipose tissue but are not characterized by a pro-inflammatory phenotype 31. PPARα and -γ agonism in macrophages has been demonstrated to have anti-inflammatory effects 26, 32-39. More specifically, treatment with tesaglitazar reduced circulating pro-inflammatory cytokines and the number of infiltrating macrophages in atherosclerotic plaques and liver in models of atherosclerosis and non-alcoholic fatty liver disease, respectively 22, 36, 37. Furthermore, macrophage-specific loss of PPARγ inhibits maturation of M2, anti-inflammatory macrophages and exacerbates obesity-associated dysmetabolism *in vivo* 26. To date, the effect of a PPARa/γ dual agonist, such as tesaglitazar, on macrophage populations in adipose tissue during obesity and dysmetabolism has not been reported. Thus, we also investigated the effects of oral and liposomal delivery of tesaglitazar on adipose tissue-resident macrophages using flow cytometry.

In summary, our approach comparing intravenous delivery of tesaglitazar in liposomes to non-liposomal, oral administration was used to (1) more thoroughly assess the cellular uptake of liposomes *in vivo*, (2) determine the impact of tesaglitazar both delivered non-liposomally and in liposomes on pro-inflammatory ATM populations and on overall metabolic effects, and (3) validate the efficacy of liposomal delivery in attenuating PPAR agonism in tissues of the RES, specifically the liver and kidney. We hypothesized that liposomal delivery of tesaglitazar would attenuate PPARα/γ agonism in the liver and kidney and reduce macrophage-induced adipose inflammation to improve overall metabolic effects.

## Materials and Methods

### Non-liposomal drug preparation

Tesaglitazar was dissolved in 0.5% carboxymethyl cellulose to a concentration of 0.35 mM. Volumes administered to mice were calculated based on body weights in order to delivery 1 μmol per kg of body weight each day. The vehicle used for non-liposomal drug treatments was 0.5% carboxymethyl cellulose administered at equal volumes to that of tesaglitazar.

### Liposome preparation and characterization

#### Liposome preparation

Liposomes were initially prepared with the remote loading attractant calcium acetate using the reverse-phase evaporation technique 40 with DSPC (phosphocholine), cholesterol and PEG-2000 DSPE at a mass ratio of 2:1:1 (phospholipids were from Avanti or Lipoid; cholesterol from Sigma). Additionally, during this step liposomes were fluorescently labeled by adding DiD lipid dye at a concentration of 1 mg DiD per 1 ml of liposomes (molar ratio of 46:1 of DSPC:DiD). DiD is an accepted abbreviation for 1,1'-dioctadecyl-3,3,3',3'tetramethylindocarbocyanine dye. As this material has two octadecyl "fatty tails" like DSPC, the main component of the liposomes, we do not expect a significant amount to be outside of the lipid membrane. Long-chain phospholipids normally possess critical micelle concentrations in the picomolar range, so we expect a negligible amount of free dye present. Lipid dyes like DiO, DiD and DiI are routunely used for liposome research and they are considered non-exchangeable 41.

Briefly, an ether-chloroform solution of lipids was mixed with aqueous calcium acetate (Ca-acetate, 1 M, pH 7.4). The ratio between organic and aqueous phase was 4:1. A mixture was subjected to emulsification by sonication (XL2020, Misonix, 50% power, 30 sec) and then organic solvents were removed under vacuum using a rotary evaporator (Re111, Buchi) connected to a vacuum line. Resulting liposomes were subjected to repeated Nuclepore filtration to achieve homogeneous size distribution, as determined by dynamic laser light scattering (DLS, Nicomp 370). External Ca-acetate was removed using a Zeba spin-column and to half of the batch, aqueous tesaglitazar in HEPES buffer (pH 7.4) was added and incubated with mixing at 37ºC for 1 hour. External unentrapped tesaglitazar was removed from liposomes with a Zeba spin-column. The vehicle used for liposomal treatments was liposomes containing aqueous calcium acetate. These were administered at volumes calculated to deliver comparable numbers of vehicle-loaded liposomes to the number of tesaglitazar-loaded liposomes per mouse.

#### Quantifying drug loading, liposome size, shape, and zeta potential

Drug loading was determined by measuring 270 nm using ultraviolet-visible spectroscopy (UV-vis). Particles per volume were quantified by Nanoparticle Tracking Analysis (Nanosight NS300, Malvern Instruments Ltd., Worcestershire, UK) in order to calculate μg of tesaglitazar per mg of DPSC lipid. Dynamic light scattering (Particle Sizing System, Inc, Santa Barbara, CA) was utilized to quantify particle size. Liposomes were also imaged using cryoTEM to assess particle structure. Additionally, zeta potential was measured using a Malvern ZetaSizer, in 10 mM HEPES buffer pH 7.4 and 25°C.

#### Liposome release kinetics

Release kinetics were determined by ultrafiltration in an Amicon 10 KDa 0.5 ml ultrafilter cartridge, where an aliquot of liposomes was added to buffer and spun to separate liposomes from the released free drug in the buffer. Concentration of tesaglitazar outside of the liposomes was quantified by UV-vis following ultrafiltration.

### Animals

Male C57Bl/6 leptin-deficient (*ob/ob*) and high-fat diet-fed C57Bl/6 (DIO) mice were purchased from Jackson Labs (Stock # 000632 and # 380050, respectively). Experiments were performed using 9- to 14-week old male *ob/ob* mice and 16-week old male DIO mice that were fed an obesity diet (60% cholesterol, Research Diets D12492) for 10 weeks. All animal experiments were performed in accordance with the Institutional Animal Care and Use Committee of the University of Virginia.

### *Ex vivo* biodistributions and blood pharmacokinetics

#### Blood pharmacokinetics

To quantify pharmacokinetics of oral administration of tesaglitazar, a single dose of tesaglitazar was administered by oral gavage. Blood was drawn at 15 min, 30 min, 1 h, 2 h, 4 h, 8 h, and 24 h post administration. Tesaglitazar levels at each of these time points were measured using LC-MS (see “Plasma and liver tesaglitazar concentrations” section of the methods). To quantify pharmacokinetics of tesaglitazar-loaded liposomes, a dose of approximately 2.5 μmol tesaglitazar/kg was administered via tail vein. Blood draws were collected at 1 min, 3 min, 5 min, 10 min, 15 min, 30 min, 1 h, 2 h, 6 h, and 24 h post-injection. Fluorescence molecular tomography (FMT) imaging was used to measure the amount of liposomes in circulation at each time point. Samples were imaged using the 680 nm laser of the FMT 4000 system (PerkinElmer, Waltham, MA). Pharmacokinetics of orally administered tesaglitazar and liposomal tesaglitazar were determined using non-compartmental analysis (NCA, Phoenix WinNonlin 8.1, Certara, NJ USA).

#### *Ex vivo* biodistribution

Tissues were harvested 4 and 24 hours post-injection. Liposome tissue biodistribution was measured using *ex vivo* FMT imaging of organs to determine the amount of DiD present in tissues. It was represented as percentage of injected dose per gram of tissue (%ID / g) and calculated by %ID / g = (Tissue Value * 100) / (Total injected dose) where the total injected dose was the sum of injected doses in instances in which treatments involved multiple injections.

### *In vivo* treatments and metabolic studies

Non-liposomal oral drug treatments were performed daily from the first to seventh day of the week-long treatment by oral gavage at a dose of 1 μmol/kg/day of tesaglitazar or an equal volume of vehicle (0.5% carboxymethyl cellulose). Vehicle treatment results are labeled as “Vehicle” and tesaglitazar treatment results as “Drug” in Figures 4 and 6. Liposomes containing tesaglitazar or vehicle (calcium acetate) were injected via the tail vein at a dose of 1 μmol/kg/day. Injections were made on the first, third or fourth, and seventh day of the weeklong treatment. For each injection, the appropriate amount of liposomes was administered to deliver 1 µmol/kg/day for the given number of days prior to consecutive treatment. This provided a dose that matches that of the non-liposomal delivery method. Vehicle-treated mice received equal numbers of liposomes to those given tesaglitazar-loaded liposomes. Results from treatments with vehicle-loaded liposomes are labeled as “Vehicle”, from tesaglitazar-loaded liposomes as “Drug”, and from non-liposome treatments as “PBS” in Figure 5 and 6. One day following the final treatment (day 8), mice were fasted for approximately 4 hours in wood chip-lined cages with water provided *ad libitum*. Following fasting, a small tail snip was made to obtain blood for measuring blood glucose levels with a glucometer (OneTouch Ultra 2 glucometer and UniStrip Technologies 24850). Mice were then placed under anesthesia (Isofluorane) and blood was collected via retro-orbital bleed. Blood was treated with EDTA (0.5 M) and spun down to collect plasma to measure insulin (ALPCO, 80-INSMR-CH01), triglyceride and glycerol levels (Sigma, TR0100), and cholesterol levels (ThermoFisher, TR13421).

### QUICKI Index calculations

To determine insulin sensitivity for each mouse, the quantitative insulin sensitivity check index (QUICKI Index) was used. The following equation is used to calculate this index: QUICKI Index = 1/[log(I0) + log(G0)] where I0 is fasting insulin in μU/mL and G0 is fasting glucose in mg/dL 42.

### Plasma and liver tesaglitazar concentrations

#### Plasma sample preparations

Plasma samples (20 µL) as well as spiked, serially diluted blank plasma (20 µL, for standard curve) were placed in a plate (Thermofisher, 260252) and 150 µL of cold acetonitrile (containing internal standard and 0.2 % formic acid) was added to each well. After mixing and centrifugation (20 min, 10,000 G at 4°C), 75 µL of the supernatant was diluted with 75 µL Milli-Q water (containing 33 % acetonitrile and 0.2 % formic acid).

#### Liver sample preparations

Frozen liver sample weights were recorded (~50-100 mg) and placed in a 2 mL tube (Sarstedt, 72.694.007) containing 6 ceramic beads (Retsch, 05.368.0090) and PBS (1x, pH 7.4) was added in a 4-fold ratio to liver sample weight. Samples were homogenized for 2x20 sec at 5000 rpm (Precellys 24, Bertin, France) and additionally 5 min at 25 Hz (Mixer Mill 301, Retsch, Germany) and centrifuged (10 min, 10,000 G at 4°C). The supernatant (50 µL) as well as spiked, serially diluted blank liver supernatant (50 µL, for standard curve) were placed in a plate (Thermofisher, 260252) and 180 µL of cold acetonitrile (containing internal standard and 0.2 % formic acid) was added to each well. After mixing and centrifugation (20 min, 10,000G at 4°C), 75 µL of the supernatant was diluted with 75 µL Milli-Q water (containing 33 % acetonitrile and 0.2 % formic acid).

#### Liquid chromatography-mass spectrometry (LC-MS)

Analysis of tesaglitazar concentrations in plasma and liver were performed using reverse phase LC-MS (UPLC Acquity coupled to a Quattro Premier XE, Waters Corporation, Milford, MA, USA). The mobile phases consisted of (A) 2% acetonitrile, 0.1% formic acid in water and (B) 0.1% formic acid in acetonitrile. Separation was performed on an Acquity UPLC HSS T3 1.8 µm column (Waters Corporation, Milford, MA, USA) with the gradient (0.7 mL/min) increased from 5-95% B over 1.0 min, held at 95% B for 1.0 min and returned to initial conditions in one step. Detection was achieved using positive electron-spray ionization (ES+) and the mass transition was 409-199 (CV: 10; CE: 21). Data acquisition and evaluation were performed using MassLynx 4.1 (Waters Corporation, Milford, MA, USA). The method showed linearity over a concentration range of 0.013-3.0 µM.

### Tissue harvest

Peritoneal lavages were collected prior to cardiac puncture to collect blood. Perfusion was performed through the left ventricle (after cutting the right atrium) with 10 mL PBS supplemented with 0.5 mM EDTA followed by 5-10 mL of PBS before harvesting all other tissues. Inguinal lymph nodes were removed before harvesting the inguinal (subcutaneous) adipose tissue. All tissues harvested for RNA extraction or cholesterol assays were flash frozen in liquid nitrogen and stored at -80°C.

### Processing tissues for flow cytometry

#### Peritoneal cells

Peritoneal cells were collected by peritoneal lavage. Lavages were spun down and treated with AKC lysis buffer (0.15 M NH_4_Cl, 0.01 M KHCO_3_, 0.1 mM EDTA) to lyse remaining red blood cells. Cells were then washed with FACS buffer (PBS, 0.05% NaN_3_, 1% BSA) to be stained for flow cytometry.

#### Adipose stromal vascular fraction (SVF) cells

Whole adipose tissue was placed in digestion buffer (0.12 M NaCl, 4.7 mM KCl, 1.3 mM CaCl_2_2H_2_O, 1.2 mM KH_2_PO_4_, 1.2 mM MgSO_4_7H_2_O, 40 mM HEPES (pH 7.5), 2.5% BSA, 200 nM adenosine, 1 mg/mL Collagenase Type 1), minced, and incubated at 37°C with shaking for 45 minutes. Digested tissue was then washed with FACS buffer, and pelleted separating floating adipocytes from the remaining stromal vascular fraction (SVF) in the pellet. Cells were treated with AKC lysis buffer to lyse remaining red blood cells and then filtered through a 70 µm filter to remove undigested tissue and/or matrix proteins. Cells were then stained for flow cytometry.

#### Bone marrow cells

Following perfusion, rear femurs and tibias were harvested and excess muscle and tissue removed. The ends of each bone were cut away to access the marrow. Using 5mL of PBS per bone, each bone was flushed using a syringe. Cell suspensions were spun and treated with AKC lysis buffer to lyse remaining red blood cells. Cells were then washed with FACS buffer to be stained for flow cytometry.

#### Spleen

Spleens were mashed through a 70 µm filter and washed with 10 mL of FACS buffer, then spun down. Cell pellets were resuspended in 5 mL of AKC lysis buffer and incubated for 5 minutes before being quenched with 5 mL of FACS buffer. Cells were then spun down and aliquoted to use 1/50^th^ of each sample for flow cytometry.

#### Blood cells

100 μL of blood was treated with AKC lysis buffer for 5 minutes. Lysis was quenched with FACS buffer and cells were spun down to be stained for flow cytometry.

### Processing livers for cholesterol assays

To quantify cholesterol levels, liver samples (50-100 mg) were homogenized in 2 ml Folch (chloroform/methanol, 2:1, v/v) with a polytron homogenizer. The organic phase was separated with 1 mL of water and centrifugation and then dried under nitrogen. Samples were reconstituted in isopropranol:Triton-X100 (9:1 v/v) and aliquots subjected to colorimetric enzymatic assays for total cholesterol (ThermoFisher, TR13421).

### Flow cytometry

All cells were stained with Live/Dead (ThermoFisher, L34966) in PBS for 30 minutes at 4°C then washed with FACS buffer. Next, the cells were stained with fluorescently-labeled antibodies against cell surface proteins (Table S1) in FACS buffer for 25 minutes at 4°C then washed with FACS buffer. Cells were then fixed with 2% PFA for 7-10 minutes at room temperature and washed with FACS buffer. If cells were sorted, they were not fixed. Finally, cells were re-suspended in FACS buffer and stored at 4°C until analyzed. Fixed samples were run on the Attune NxT (Thermofisher; one-week liposome uptake experiments) or CyAN ADP LX (Beckman Coulter; 4 hour and 24 hour liposome uptake and one-week macrophage subset experiments) and live cells were sorted on the INFLUX (BD).

### Real-time polymerase chain reaction

RNA was extracted from tissues and cells using Trizol extraction. One µg of RNA was then treated with DNase (Invitrogen) and used to reverse transcribe cDNA using an iScript cDNA synthesis kit (BioRad). To quantify gene expression, cDNA was diluted 1:10 in water and combined with 0.5 mM forward and reverse primers (Table S2) and SYBR Green (SensiFast, BioLine). Semi-quantitative real-time PCR was performed on a CFX96 Real-Time System with an annealing temperature of 60°C for all reactions (BioRad). Data were calculated by the ΔΔCt method and expressed in arbitrary units that were normalized to 18s ribosomal RNA or Tata box binding protein (Tbp) levels.

### Immunofluorescence

Livers were fixed in 4% PFA and then subjected to a sucrose gradient (10% overnight, 20% 6hrs, 30% overnight) at 4°C, rotating. Then, tissues were embedded in OCT and 10 μm sections obtained. For staining, tissue sections were permeabilized with 0.25% Triton-100 in PBS, and then washed in PBS. Sections were blocked with 10% Horse Serum in 0.3% fish skin gelatin in PBS, then incubated with rat anti-CLECSF13 antibody (R&D Systems, MAB2784) at a 1:250 dilution in 10% Serum in PBS overnight at 4°C. Sections were washed as before and then incubated with donkey anti-rat Dylight 550 secondary antibody at a 1:250 dilution. Following one final wash, slides were counterstained with DAPI and coverslipped using ProLong Gold (Life Technologies). Z-stack images were obtained at 1 μm intervals using Zeiss LSM700 confocal microscope, 20X objective. Figures shown are maximal intensity projection images.

### Whole-mounted imaging

Aliquots of epididymal and subcutaneous adipose were fixed in 4% PFA then washed in PBS. Adipose was blocked and permeabilized in 5% BSA, 0.3% Triton in PBS before incubating overnight with an anti-CD68-PE conjugated antibody (Biolegend, Clone FA-11) and an Isolectin GS-IB_4_-AF488 conjugate (Thermofisher) at 4°C. After a final wash, samples were mounted in a 1:1 solution of PBS:Glycerol and digital images were acquired using confocal microscopy (Nikon Instruments Incorporated, Model TE200-E2; 20X objective). 40 μm Z-stacks with 2 μm step size were acquired with a 20x magnification power and processed using ImageJ software.

### Statistics

All statistical analyses were performed using Prism 7 (GraphPad Software, Inc.). Mann-Whitney U tests were used to analyze oral treatment experimental groups, while Kruskal-Wallis with Dunn's multiple comparison tests were used to analyze liposome treatment experimental groups. Data are expressed as mean ± standard deviation (SD). P values are specified in figure legends.

## Results

### Synthesis, circulation kinetics, and tissue biodistribution of tesaglitazar-loaded liposomes

**Figure 1:**
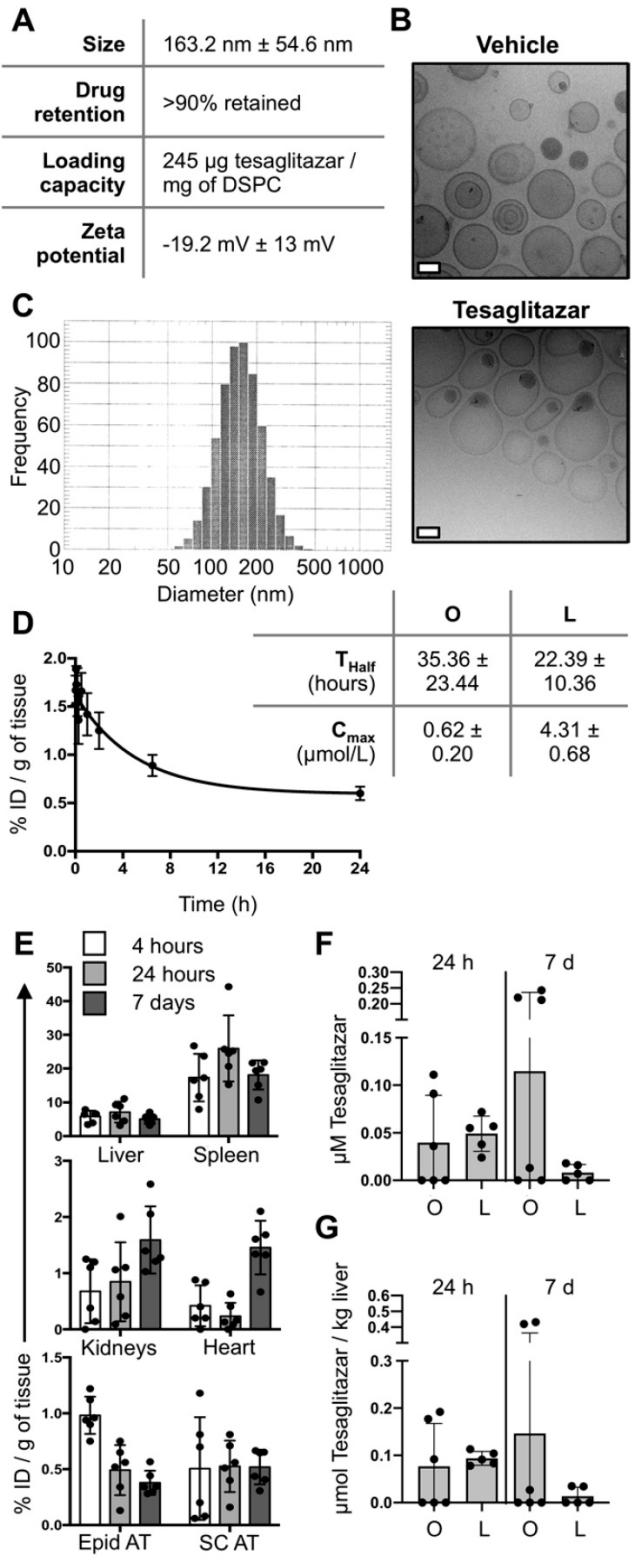
** Liposome synthesis and blood and tissue PK and biodistribution. (A)** Liposomes were synthesized, labeled with DiD, and loaded with tesaglitazar. Data reporting liposome characteristics are listed. **(B)** CryoTEM images of vehicle- and tesaglitazar-loaded liposomes are displayed to provide examples of liposome shape. White scale bars represent 50 nm. **(C)** DLS was utilized to quantify liposome size. **(D)** Tesaglitazar was administered orally (O) and in DiD-labeled liposomes (L) and blood was harvested at multiple time points to calculate the half-life (T_half_) and C-max (C_max_) of drug in circulation using non-compartmental analysis. **(E)** FMT was used to quantify liposome uptake in liver, spleen, kidneys, heart, Epid and SC adipose tissues four and 24 hours following administration as well as after seven days with three administrations of liposomes. **(F,G)** LC-MS was utilized to quantify tesaglitazar levels in circulation (F) and in liver tissue (G) at 24-hour and 7-day time points post-treatment. Standard oral formulation (O) and liposomal (L) delivery methods were compared to verify comparable drug exposure levels. Data represents the mean ± SD.

PEGylated liposomes labeled with fluorescent DiD and loaded with tesaglitazar were synthesized for these studies with an average size of approximately 160 nm (Figure 1A-C). Following months of refrigerated storage, particle size and size distribution did not change significantly (Figure 1A). Repeats of the ultrafiltration tests showed that tesaglitazar was not presented at a significant quantity outside the ultrafilters, with over 90% retained. This drug retention is similar to what is observed for Doxil/Lipodox, which is also prepared by remote loading (Figure 1A). The zeta potential of the liposomes was -19.2 mV ± 13 mV and drug loading was 245 μg/mg of DSPC (Figure 1A). Pharmacokinetic and biodistribution studies were performed using LC-MS and fluorescence molecular tomography (FMT) and the amount of DiD in each tissue was quantifed from reconstructed images. The half-life of the liposomes in circulation was estimated to be 22.4 ± 10.4 h by non-compartmental analysis. It is hence in the same order of magnitude as the half-life of orally delivered tesaglitazar (35.4 ± 23.4 h, Figure 1D). Estimations of the C-max reveal a value of 0.62 ± 0.20 μmol tesaglitazar/L for orally-delivered tesaglitazar, while liposomal delivery demonstrated a C-max value of 4.38 ± 0.68 (Figure 1D).

Calculated as percent injected dose per gram of tissue, DiD content in the liver, spleen, kidney, heart, epididymal (Epid) and subcutaneous (SC) adipose tissues were quantified at 4 hours, 24 hours and 7 days post-liposome treatment (Figure 1E). Levels of tesaglitazar at 24 hour and 7 days post-treatment in circulation and the liver were quantified using LC-MS and revealed that standard oral formulation and liposome treatments resulted in similar drug accumulation in both compartments (Figure 1F,G). It should be noted that neither of these time points were at C-max.

### Macrophages were the predominant cell type that took up liposomes in visceral white adipose tissue

**Figure 2:**
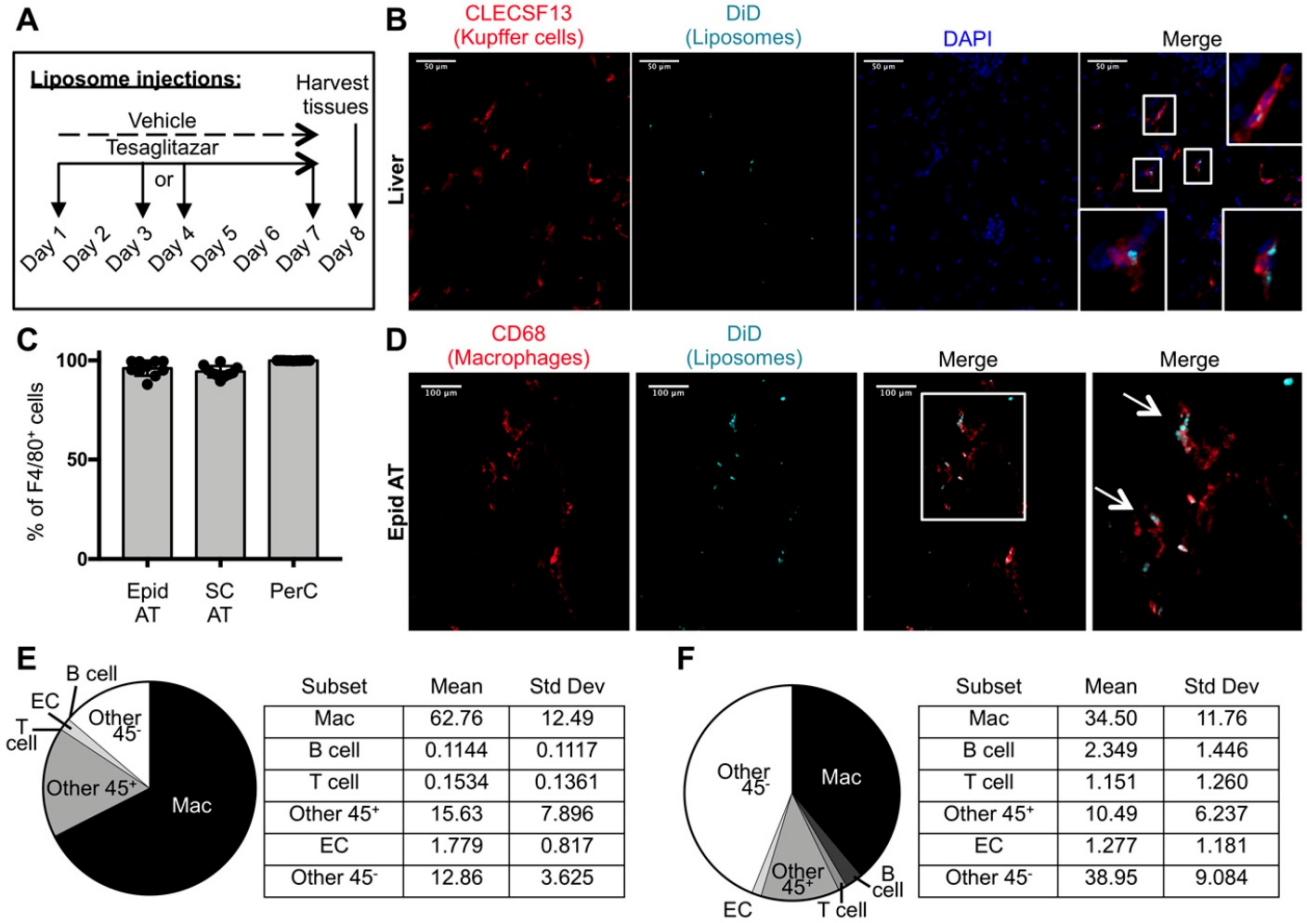
** Cellular characterization of liposome uptake after one week of treatment. (A)** DiD-labeled liposomes were injected intravenously into male *ob/ob* mice three times over the course of seven days. **(B)** Z-stack images of liver sections from *ob/ob* mice treated with tesaglitazar-loaded liposomes were stained with CLECS13F to identify Kupffer cells and assessed for co-localization with DiD-labeled liposomes. Co-localization of CLECS13F^+^ cells and DiD are marked by white boxes. **(C)** Peritoneal lavages and Epid and SC AT were harvested to stain peritoneal cavity (PerC) cells and SVF cells, respectively, for analysis by flow cytometry. The percentage of CD45^+^F4/80^+^ macrophages that were DiD^+^ was quantified. **(D)** Z-stack images of whole mounted Epid AT from an *ob/ob* mouse treated with tesaglitazar-loaded liposomes was stained with CD68 to identify macrophages and assessed for co-localization with DiD-labeled liposomes. Co-localization of interstitial CD68^+^ cells and DiD are marked by white arrows. The white box delineates the area of the merged image that is enlarged (right-most panel). **(E,F)** DiD^+^ macrophages and other cell subsets were also quantified as a percent of total DiD^+^ cells in the Epid AT (E) and SC AT (F), n = 5 in each group. The cell subsets analyzed were macrophages (Mac, CD45^+^F4/80^+^), B cells (CD45^+^CD19^+^), T cells (CD45^+^CD3^+^), other CD45^+^ Cells (Other 45^+^, CD45^+^CD19^-^CD3^-^F4/80^-^), endothelial cells (EC, CD45^-^CD31^+^), and other CD45^-^ cells (Other 45^-^, CD45^-^CD31^-^). Data represents the mean ± SD.

To characterize the cell types that take up drug-loaded liposomes in our system, DiD-labeled liposomes were administered intravenously to *ob/ob* mice three times over the course of one week (Figure 2A). Immunofluorescence staining of livers from these mice revealed co-localization of DiD and Kupffer cell marker CLECSF13 (Figure 2B, Figure S1). Flow cytometry was performed to identify the cell types that take up tesaglitazar-loaded liposomes in adipose tissue and the peritoneal cavity (Figure S2A, Figure S3A). We found that nearly all CD45^+^F4/80^+^ macrophages in the adipose stromal vascular fraction (SVF) and the peritoneal cavity (PerC) were DiD^+^ (Figure 2C). Consistent with flow cytometry findings, immunofluorescent staining with the macrophage marker CD68 demonstrated that DiD-labeled liposomes co-localized with macrophages within whole mounted white adipose tissue samples (Figure 2D, Figure S4). Of the total DiD^+^ population, macrophages made up approximately 67% and 40% of DiD^+^ cells in epididymal (Figure 2E) and subcutaneous (Figure 2F) SVFs, respectively. Other CD45^+^ cells as well as CD45^-^ vascular and stromal cells, particularly endothelial cells (ECs), were DiD^+^ demonstrating that cells other than professional phagocytes are capable of liposomal uptake (Figure 2E-F, Figure S2B,C). Various cell types including macrophages and other immune cells such as B and T cells in the peritoneal cavity (Figure S3B), bone marrow (Figure S5B), and blood (Figure S6B) also took up liposomes.

**Figure 3:**
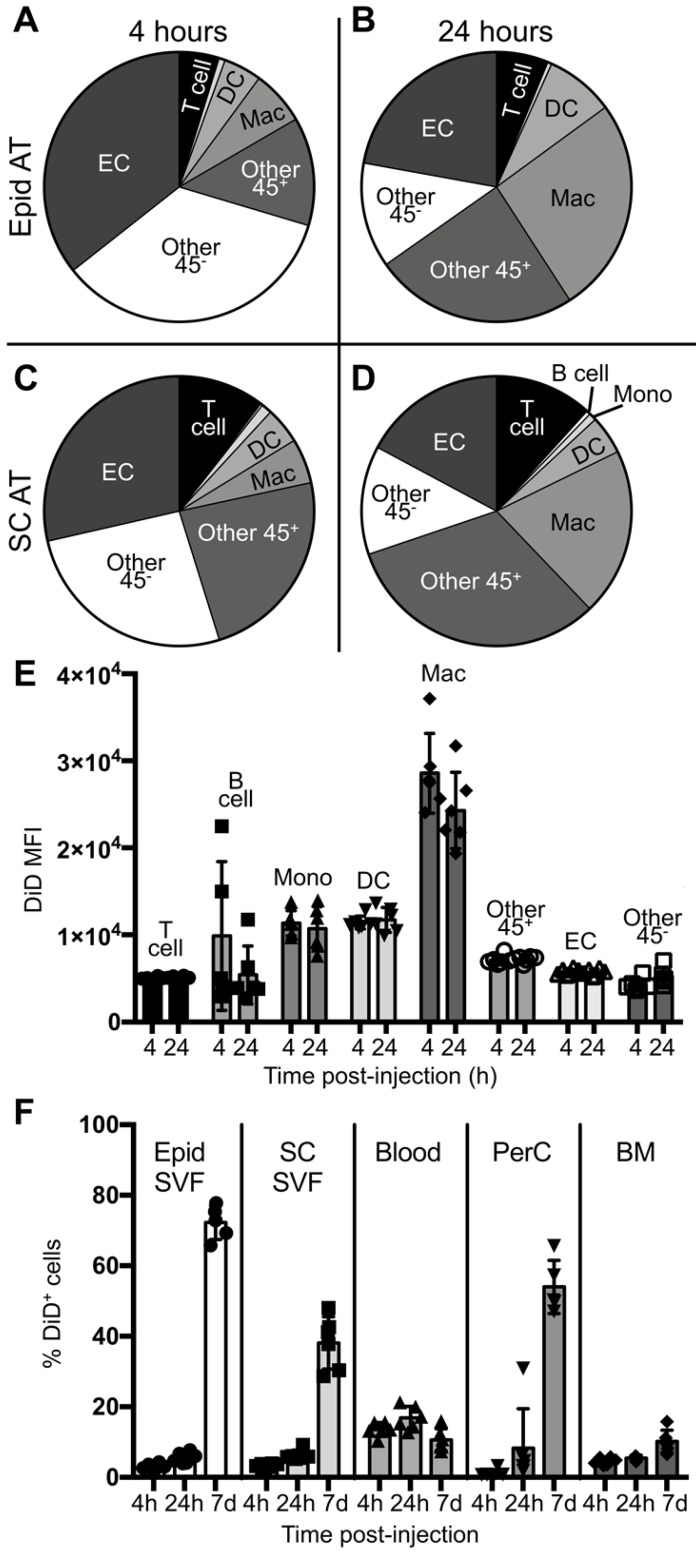
** Cellular biodistribution of liposomes at 4- and 24-hour time points.** DiD-labeled liposomes were injected intravenously into male *ob/ob* mice and tissues were harvested four or 24 hours later. Peritoneal lavages, bone marrow, blood and Epid and SC AT were harvested to stain peritoneal cavity (PerC), bone marrow (BM), blood, and SVF cells, respectively, for analysis by flow cytometry. **(A-D)** DiD^+^ macrophages and other cell subsets were also quantified as a percent of total DiD^+^ cells at four **(A,C)** and 24 hours **(B,D)** post-injection in the Epid AT (A,C) and SC AT **(B, D)**, n = 6 in each group. The cell subsets analyzed were macrophages (Mac, CD45^+^F4/80^+^), B cells (CD45^+^CD19^+^), T cells (CD45^+^CD3^+^), monocytes (Mono, CD45^+^CD115^+^), dendritic cells (DC, CD45^+^CD11c^+^), other CD45^+^ Cells (Other 45^+^, CD45^+^CD19^-^CD3^-^F4/80^-^ CD11c^-^ CD115^-^), endothelial cells (EC, CD45^-^CD31^+^), and other CD45^-^ cells (Other 45^-^, CD45^-^CD31^-^). **(E)** The mean fluorescence intensity (MFI) of DiD within each of these subsets was also quantified in Epid AT. **(F)** The percent of total cells in all aforementioned tissues that were DiD^+^ was also quantified. Data represents the mean ± SD.

To better understand the initial kinetics by which liposomes are taken up by macrophages and other cell types, male *ob/ob* mice were administered a single dose of tesaglitazar-loaded DiD-labelled liposomes and the circulation time and cellular uptake of liposomes at 4 and 24 hours post-injection was assessed. A significant proportion of CD115^+^ monocytes, which can differentiate into macrophages, in the blood were DiD^+^ at 4 and 24 hours post-injection (Figure S6C). Additionally, nearly all macrophages found in the spleen were also DiD^+^ at early time points (Figure S7) and within the bone marrow, macrophages make up the highest proportion of DiD^+^ cells (Figure S5D). Within the adipose, a smaller proportion of macrophages were DiD^+^ and, notably, CD31^+^ ECs and other CD45^-^ stromal and vascular cells made up a greater proportion of DiD^+^ cells after four and 24 hours (Figure 3A-D, Figure S8). However, when quantifying the DiD mean fluorescent intensity (MFI), which is the level of fluorescence per cell, DiD MFI was highest in the macrophage population in adipose tissue suggesting that macrophages took up a larger portion of liposomes per cell than other subsets (Figure 3E, Figure S2D). When comparing uptake at four hours post-injection to 24 hours post-injection, an increase in the proportion of macrophages that were DiD^+^ can already be observed (Figure S2E). This accumulation of liposomes in the adipose tissue as well as the peritoneal cavity continues over the course of a seven-day treatment (Figure 3F).

### Tesaglitazar delivered as a standard oral formulation improved metabolic parameters and reduced total macrophage and pro-inflammatory macrophage numbers in white adipose tissue

**Figure 4:**
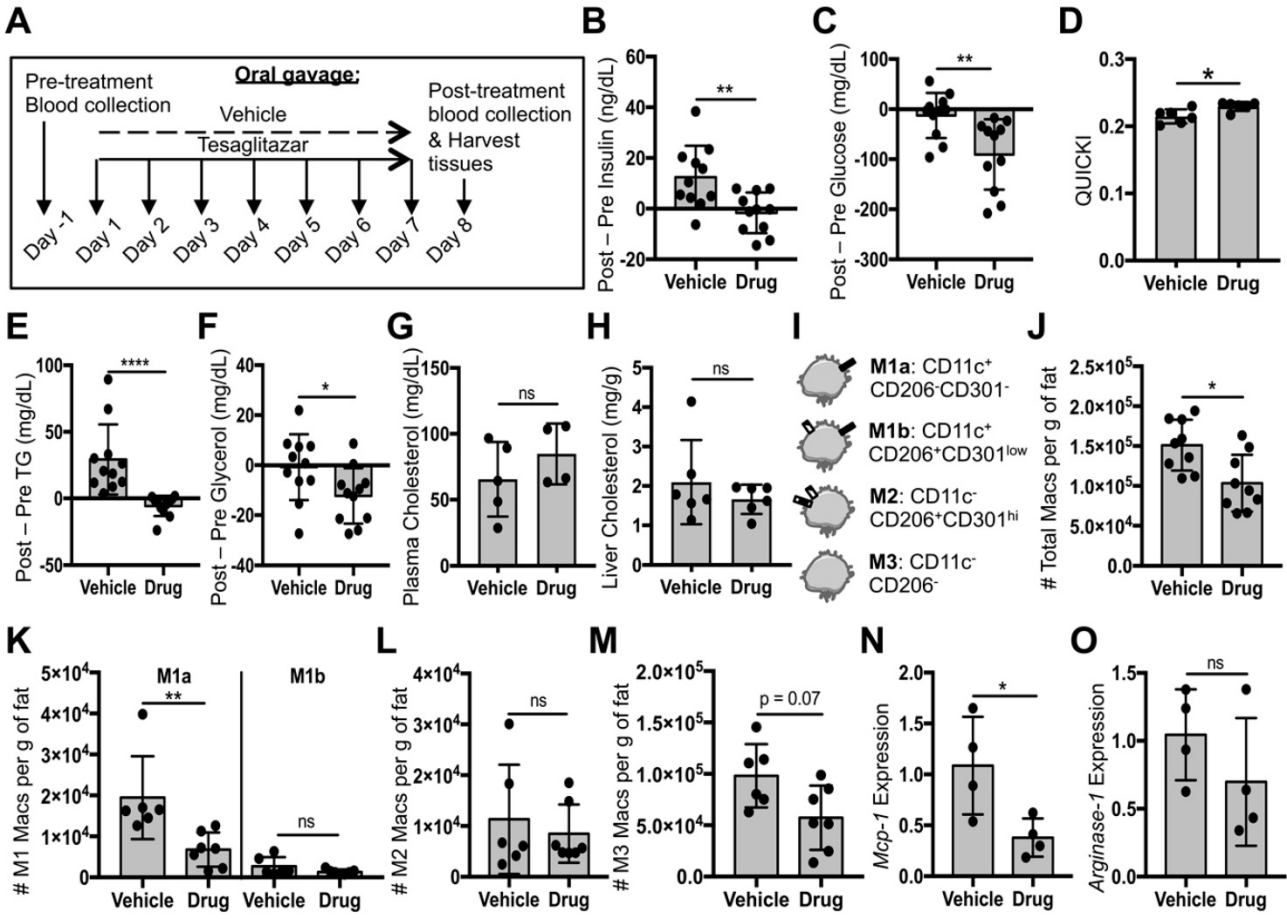
** Tesaglitazar delivered as a standard oral formulation improved metabolic parameters and reduced total macrophage and pro-inflammatory macrophage numbers in white adipose tissue. (A)** Male *ob/ob* mice were treated daily by oral administration of tesaglitazar or vehicle for seven days. To assess metabolic effects, blood was harvested from mice before and after treatments and plasma was harvested. **(B,C)** Circulating insulin **(B)** and glucose **(C)** levels before and after treatment were measured and the changes in levels per mouse were calculated. **(D)** Post-treatment levels were also utilized to quantify QUICKI index for each mouse. **(E-G)** Circulating triglyceride **(E)**, glycerol **(F)**, and cholesterol **(G)** levels before and after treatment were measured and the changes in levels per mouse were calculated. **(H)** Post-treatment cholesterol levels in the liver were also quantified. **(I)** Epid SVF cells from *ob/ob* mice were stained with antibodies against markers of macrophages and macrophage subsets to quantify cell numbers by flow cytometry. **(J)** Total CD45^+^CD11b^+^F4/80^+^ macrophage numbers from epididymal adipose were normalized to the total mass of the adipose depot. **(K-M)** M1a and M1b **(K)**, M2 **(L)**, and M3 **(M)** macrophage subsets were quantified and normalized to total adipose mass as well. **(N,O)** RNA was extracted from sorted CD45^+^CD11b^+^F4/80^+^ peritoneal macrophages and macrophage chemokine *Mcp-1* **(N)** and M2 marker *Arginase-1* **(O)** expression levels were quantified. Data represents the mean ± SD; * p ≤ 0.05, ** p ≤ 0.01, **** p ≤ 0.0001. Vehicle indicates animals treated orally with vehicle, drug indicates animals treated orally with tesaglitazar.

Given the established anti-inflammatory role of PPARα and -γ in macrophages, we first investigated whether tesaglitazar administered as a standard oral formulation had effects on macrophage populations *in vivo*. *Ob/ob* mice were treated with tesaglitazar via daily oral administration for one week before assessing circulating metabolic parameters and ATM populations by flow cytometry (Figure 4A). Consistent with previous studies 43, we found that one week of oral tesaglitazar treatments reduced levels of circulating insulin (Figure 4B) and glucose (Figure 4C). These changes resulted in improved indices of insulin sensitivity, as indicated by the QUICKI index (Figure 4D). Tesaglitazar treatment also resulted in reduced triglycerides (Figure 4E) and glycerol (Figure 4F), but no change in cholesterol levels in circulation (Figure 4G) or in the liver (Figure 4H). Efficacy of tesaglitazar treatment in a model of high-fat diet-induced obesity was performed to validate efficacy of the treatment in other models of obesity-associated dysmetabolism. One week of daily tesaglitazar treatments effectively lowered circulating insulin (Figure S9A) and triglyceride levels (Figure S9B), but did not affect glucose (Figure S9C) or glycerol levels (Figure S9D), nor did it improve QUICKI index values (Figure S9E). Furthermore, we found that equal doses of tesaglitazar for one week did not induce expression of PPARα and -γ gene targets in the liver and Epid AT as effectively in the DIO model as they did in the *ob/ob* strain of mice (Figure S9F). For these reasons, the *ob/ob* model was utilized in all other experiments in this study.

Flow cytometry gating for macrophage subsets in this study was based on previously published strategies 31, 44, 45 (Figure 4I, Figure S2A). Mice treated orally with tesaglitazar had fewer total CD45^+^F4/80^+^CD11b^+^ macrophages in Epid AT (Figure 4J) with a trend to fewer macrophages in SC AT (Figure S10B). Additionally, the standard oral formulation of tesaglitazar reduced pro-inflammatory M1a macrophage numbers (Figure 4K, Figure S10C), but did not change pro-inflammatory M1b (Figure 4K, Figure S10C) or resident, anti-inflammatory, M2 macrophage numbers (Figure 4L, Figure S10D). There was also a trend towards reducing the number of Epid M3 macrophage numbers (Figure 4M), which have been characterized as macrophages enriched in mRNA expression of chemokine receptors *Ccr9*, *Ccr2*, and *Cx3cr1* 46. Finally, we measured expression of M1 gene marker *Monocyte chemoattractant protein-1* (*Mcp-1*) and M2 gene marker *Arginase-1* in CD45^+^CD11b^+^F4/80^+^ macrophages sorted from mice treated with vehicle or tesaglitazar and found that standard oral formulation treatments of tesaglitazar reduced *Mcp-1* expression levels (Figure 4N), but did not affect *Arginase-1* levels (Figure 4O).

### Liposomal delivery of tesaglitazar does not substantially improve metabolic parameters nor reduce pro-inflammatory ATM numbers

**Figure 5:**
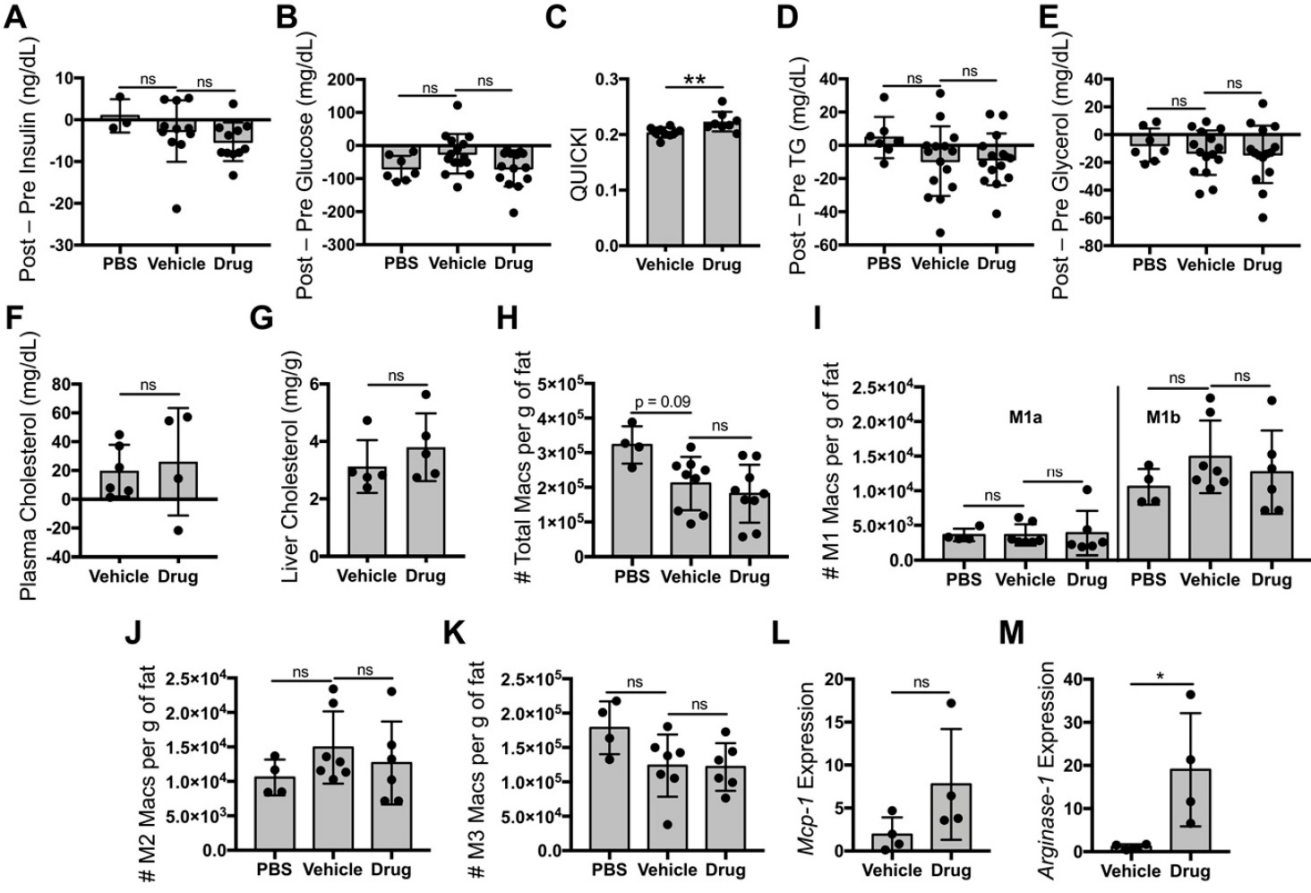
** Liposomal delivery of tesaglitazar does not improve metabolic parameters nor reduce pro-inflammatory ATM numbers.** Male *ob/ob* mice were treated intravenously three times over the course of seven days with PBS, vehicle-loaded liposomes, or tesaglitazar-loaded liposomes. To assess metabolic effects, blood was harvested from mice before and after treatments and plasma was harvested. **(A,B)** Circulating insulin (A) and glucose (B) levels before and after treatment were measured and the changes in levels per mouse were calculated. **(C)** Post-treatment levels were also utilized to quantify QUICKI index for each mouse. **(D-F)** Circulating triglyceride (D), glycerol (E), and cholesterol (F) levels before and after treatment were measured and the changes in levels per mouse were calculated. **(G)** Post-treatment cholesterol levels in the liver were also quantified. As before, Epid SVF cells from *ob/ob* mice treated via oral or liposomal delivery for seven days were stained with antibodies against markers of macrophages and macrophage subsets to quantify cell numbers by flow cytometry. **(H)** Total CD45^+^CD11b^+^F4/80^+^ macrophage numbers from epididymal adipose were normalized to the total mass of the adipose depot. **(I-K)** M1a and M1b (I), M2 (J), and M3 (K) macrophage subsets were quantified and normalized to total adipose mass as well. **(L,M)** RNA was extracted from sorted CD45^+^CD11b^+^F4/80^+^ peritoneal macrophages and macrophage chemokine *Mcp-1* (L) and M2 marker *Arginase-1* (M) expression levels were quantified. Data represents the mean ± SD; * p ≤ 0.05, ** p ≤ 0.01. Vehicle indicates vehicle-loaded liposomes, drug indicates tesaglitazar-loaded liposomes, PBS indicates no liposomes.

Since most ATMs took up liposomes, we then investigated whether liposomal delivery of tesaglitazar (Figure 2A) would affect metabolic parameters and ATM populations similarly to that of orally administered tesaglitazar (Figure 4A). One week of treatment with tesaglitazar-loaded liposomes did not significantly lower levels of fasting blood insulin (Figure 5A) or glucose (Figure 5B), but did improve indices of insulin resistance (Figure 5C). Tesaglitazar treatments did not reduce triglycerides (Figure 5D), glycerol (Figure 5E), or cholesterol in circulation (Figure 5F) in the liver (Figure 5G) beyond those of vehicle-loaded liposomes. Additionally, liposomal delivery of tesaglitazar did not affect total macrophage numbers in Epid or SC AT (Figure 5H, Figure S10B) compared to vehicle liposomes. Tesaglitazar-loaded liposomes did not alter M1a (Figure 5I, Figure S10C), M1b (Figure 5I, Figure S10C), M2 (Figure 5J, Figure S10D), or M3 (Figure 5K, Figure S10E) macrophage numbers compared to vehicle-loaded liposomes or PBS. Interestingly, liposomal delivery of tesaglitazar did not affect M1 gene marker *Mcp-1* (Figure 5L), but induced M2 gene marker *Arginase-1* (Figure 5M) in sorted CD45^+^CD11b^+^F4/80^+^ macrophages.

### Liposomal delivery of tesaglitazar attenuates PPARα/γ agonism in the liver and kidney

**Figure 6:**
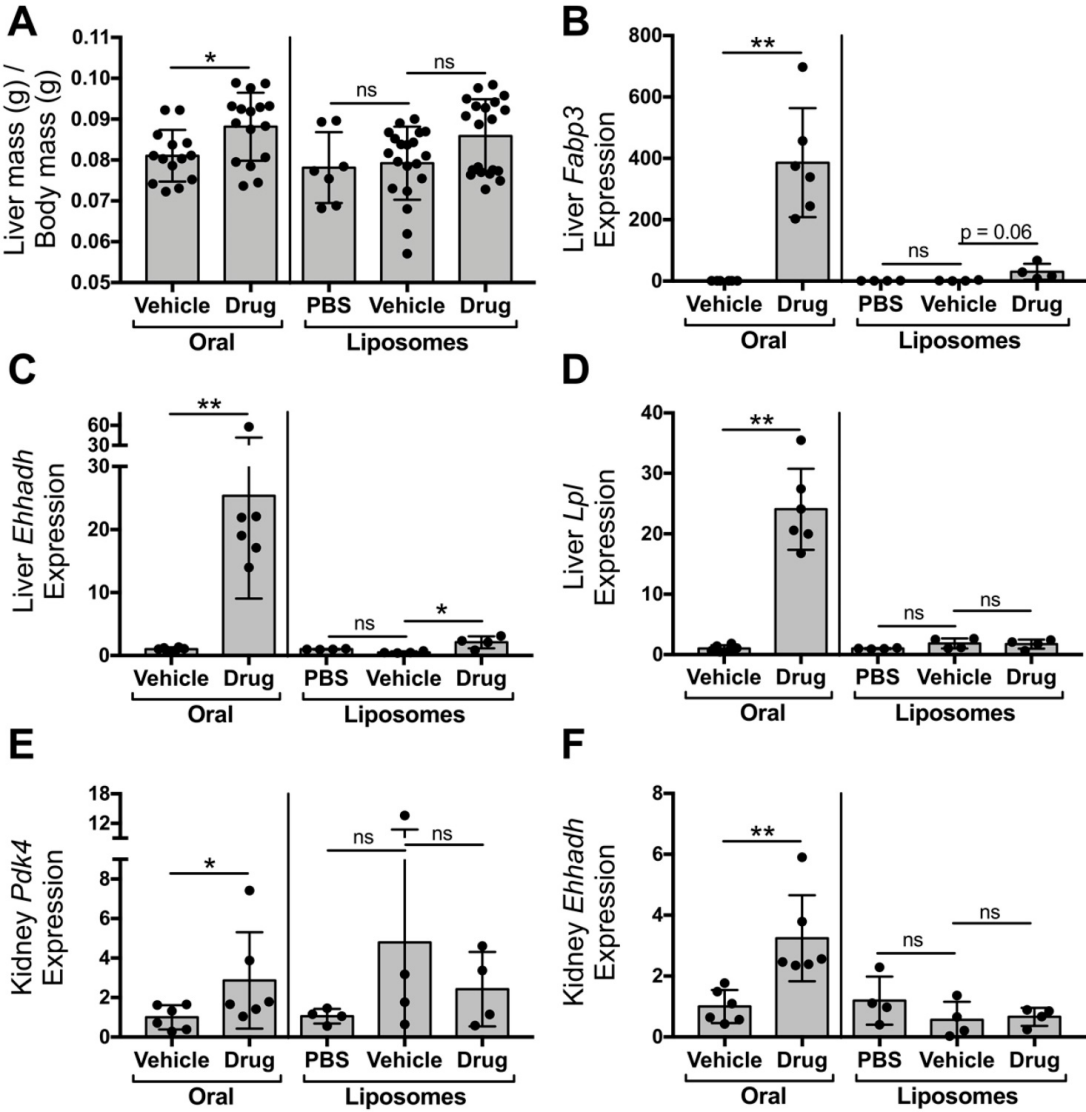
** Liposomal delivery attenuates tesaglitazar-induced effects in the kidney and liver observed with administration of a standard oral formulation.** Male *ob/ob* mice were either treated daily by oral administration of tesaglitazar or vehicle for seven days or treated intravenously three times over the course of seven days with PBS, vehicle-loaded liposomes, or tesaglitazar-loaded liposomes. After the final treatment, livers and kidneys were harvested from each mouse and total body and liver mass was measured. **(A)** Liver mass was quantified relative to total body mass. **(B-D)** RNA extracted from livers of each mouse was used to quantify relative gene expression of *Fabp3* (B), *Ehhadh* (C), and *Lpl* (D) by qRT-PCR. **(E-F)** RNA extracted from kidneys of each mouse was used to quantify relative gene expression of *Pdk4* (E) and *Ehhadh* (F), by qRT-PCR. Data represents the mean ± SD; * p ≤ 0.05, ** p ≤ 0.01. Vehicle indicates vehicle-loaded liposomes or vehicle-treated animals, drug indicates animals treated by tesaglitazar-loaded liposomes or a standard oral formulation, PBS indicates no liposomes.

To compare the impact of oral and liposomal delivery methods on inducing drug action in the liver and kidney, *ob/ob* mice were treated with tesaglitazar and vehicle controls by either oral (Figure 4A) or liposomal (Figure 2A) delivery. Oral delivery of tesaglitazar significantly increased liver mass in *ob/ob* mice, while there was no statistically significant change in liver mass in mice treated with tesaglitazar-loaded liposomes compared to controls (Figure 6A). Oral administration of tesaglitazar induced expression of PPARα target genes in the liver with a 386-, 25-, and 24-fold induction of *Fapb3* (Figure 6B), *Ehhadh* (Figure 6C), and *Lpl* (Figure 6D), respectively, over vehicle controls. Gene expression changes in the liver were attenuated when tesaglitazar was administered in liposomes as opposed to standard oral formulation. Indeed, liposomal delivery of tesaglitazar had no effect on *Lpl* expression in the liver (Figure 6D), and moderately induced expression of the other PPARα gene targets *Ehhadh* and *Fabp3* by 4- and 17-fold, respectively, over controls (Figure 6B,C). A similar effect was seen in the kidney: tesaglitazar induced a 3-fold increase in expression of PPARγ target genes *Pdk4* (Figure 6E) and *Ehhadh* (Figure 6F), in the kidneys compared to vehicle controls. Liposomal delivery of tesaglitazar did not significantly induce expression of PPARγ gene targets* Ehhadh* and *Pdk4* in the kidney over controls (Figure 6E,F).

## Discussion

In this study, a murine model of obesity-associated dysmetabolism was treated with PPARα/γ dual agonist tesaglitazar as a standard oral formulation or intravenously in liposomes in order to (1) further characterize the cell types that take up liposomes *in vivo*, (2) investigate whether liposomes could effectively attenuate drug action in the liver and kidney, and (3) determine if tesaglitazar delivered either as a standard oral formulation or in liposomes had anti-inflammatory effects on macrophage populations.

Ergen *et al*. recently found that myeloid subsets including macrophages take up liposomes in a number of tissues including liver, kidney, and lung, but uptake in the adipose tissue and by non-myeloid cells was not determined 47. Our results represent a fuller characterization of *in vivo* liposomal uptake in myeloid and non-myeloid cell types and include analysis of adipose tissue at multiple time points. After one week of treatment, liposomes were taken up by nearly 100% of macrophages in adipose tissue and the peritoneal cavity. With regard to potentially treating macrophage-induced effects in adipose tissue, our data would suggest that excellent delivery could be achieved. But many other cell types including CD19^+^ B cells, CD3^+^ T cells, and CD31^+^ ECs, and other CD45^-^ cells, which could be fibroblasts, vascular smooth muscle cells (VSMCs), or progenitor cells, also took up these liposomes in adipose tissue of obese mice. This finding introduces an important caveat of our study, as well as many other studies employing the use of liposomes, that may affect our understanding of the mechanisms driving observed biological outcomes. Therefore, we cannot conclude with certainty that observed tesaglitazar-loaded liposome-induced biological effects were due to uptake and action in macrophages only since so many other cell types took up liposomes.

This finding raises interesting questions regarding the cell-specific effects of tesaglitazar in non-macrophage cells. Generally, PPARα and PPARγ have anti-inflammatory effects in non-macrophage immune cells. PPARγ and -α agonism in T cells inhibits proliferation and cytokine expression 48-51 and PPARγ also promotes FoxP3^+^ Treg accumulation in adipose tissue 52, 53. PPARγ activation in dendritic cells attenuates toll-like receptor activation and promotes cell migration to lymph nodes during lung inflammation 54, 55. PPARγ activation has been shown to boost memory responses in B cells through antibody production and differentiation 56, 57. These responses may be considered beneficial or detrimental depending on the disease or infection context. In vascular and stromal cells, PPARα and -γ generally play anti-fibrotic and anti-proliferative roles. Global knockout of PPARγ in mice is embryonically lethal due to vascular defects 58 and, depending on the environment; PPARγ can be pro- or anti-angiogenic in adults 59, 60. PPARα and PPARγ both inhibit proliferation and promote apoptosis of VSMCs 61, 62 and induce anti-fibrotic effects in fibroblasts 63-65 and liver stellate cells 66. Overall, studies suggest the possibility that PPARα/γ agonism in most cells targeted by our tesaglitazar-loaded liposomes could induce anti-inflammatory responses. Whether these changes facilitate improved metabolic outcomes is unclear from this study, but the reduced PPARα and -γ agonism in the liver suggests these improvements would be limited. Metabolic changes may have also been limited due to the short duration of the treatments performed. Follow-up studies extending the duration of liposomal treatment would address whether longer-term liposome treatments could improve metabolic outcomes. Furthermore, there is the challenge of identifying the cell types in which PPARα/γ agonism has beneficial effects. Delivery methods that target specific cell types would be the optimal approach to assess this.

Additionally, assessment of liposomal uptake at different time points revealed that monocytes in circulation and vascular cells in adipose tissue initially take up liposomes. However, within the first 24 hours, the percentage of adipose SVF cells that contain liposomes significantly increases even as the MFI, or amount of DiD in the cell, does not. This suggests that it may be the cells rather than the liposomes alone that are entering from the circulation with time. These data suggest that penetration of the vessel wall by liposomes to effect drug delivery of immune cells in inflamed tissues may be facilitated by disease-associated increases in tissue immune cell accumulation. These findings prompt additional questions regarding liposome biodistribution over time and the specificity of liposome targeting that could be pursued in future studies. Overall, our fulsome approach for evaluating liposome uptake *in vivo* with flow cytometry is a useful tool to understand the cellular mechanisms by which liposomal delivery of compounds may influence biological outcomes and the residual potential risks of off target effects.

Additionally, we were able to validate that liposomal delivery of a drug can indeed attenuate drug action in liver and kidney observed by non-liposomal delivery methods. We observed equivalent drug levels in the liver at the end of treatment and greater C-max values in mice treated with liposomes than orally-delivered tesaglitazar. Furthermore, our findings suggest drug-loaded liposomes were predominantly taken up by Kupffer cells, not hepatocytes. We hypothesize that this transfer of drug delivery away from hepatocytes and toward Kupffer cells is a likely mechanism for the dampened effects in the liver and kidney. Indeed, Ehhadh is expressed at high levels in hepatocytes 67 and we observed significant attenuation of tesaglitazar-induced *Ehhadh* by liposomal delivery. Consistent with the study by Nakashiro *et al.* 25, we also found attenuation of drug-induced gene expression in the kidney. Hepatic and renal toxicity are caused by many medications and supplements, so liposomal delivery may be a valuable approach to reduce hepatocyte and kidney uptake and toxicity of many compounds.

On the other hand, some therapeutic effects of drugs are dependent on drug action in hepatocytes. Tesaglitazar is one example of such compounds: its beneficial effects are in part due to action in the liver. Indeed, loss of PPARγ expression in the liver exacerbates dysmetabolism associated with obesity 68. Our results demonstrate that tesaglitazar delivered as a standard oral formulation effectively improves indices of insulin resistance and lowers circulating levels of triglycerides, insulin, and glucose. Liposomal treatment, however, only improved QUICKI index values leaving circulating levels of triglycerides, insulin, and glucose unchanged. This reduced delivery to hepatocytes may explain the discrepancies between delivery methods on metabolic efficacy. Given the altered delivery kinetics and reduced bioavailability of liposomes while in circulation, it is also possible that longer treatment duration may result in improved metabolic effect. An additional follow-up study extending treatment duration would directly address this possibility.

The rationale for selecting tesaglitazar for this study came in two parts. Our first motive was its profound and easily quantified effects in the liver and kidney. Additionally, however, utilizing tesaglitazar provided an opportunity to test whether delivery of a PPARα/γ dual agonist to macrophages was sufficient to improve metabolic outcomes in a murine model of obesity. A study by Odegaard *et al.* demonstrated that expression of PPARγ in macrophages is important for improving insulin resistance during metabolic syndrome 26. Our approach eliminated any caveats that come with genetic knockout studies and also applied a potential therapeutic strategy to previous findings regarding the role of PPARα and -γ in macrophages.

Results of the present study are the first to fully characterize the effects of PPARα/γ dual agonism on macrophage subtypes in adipose tissue during obesity. Additionally, we employed a recently updated flow cytometry staining and gating strategy to characterize adipose tissue macrophages subtypes based on work by Lumeng and others 31. Pro-inflammatory, M1 macrophages are important cellular mediators of inflammation and insulin resistance 29, 30 and PPARα and -γ agonism and genetic knockout demonstrate anti-inflammatory effects in macrophages both *in vitro* and *in vivo* 26, 32-35. Oral administration of tesaglitazar significantly reduced the number of pro-inflammatory M1a ATMs. Consistent with the role of these subtypes in inflammation and the link between inflammation and metabolism, oral delivery of tesaglitazar also improved metabolism.

However, liposomal delivery of tesaglitazar did not reduce pro-inflammatory ATM numbers suggesting PPARα/γ dual agonism in non-macrophage cell types may influence ATM biology. The attenuated effects were not due to inadequate liposomal uptake as 95-100% of macrophages in SC AT, Epid AT, and the peritoneal cavity took up drug-loaded liposomes. Furthermore, increased PPARγ gene target *Arginase-1* levels in macrophages treated with tesaglitazar-loaded liposomes provided evidence that drug was released and PPARγ agonism occurred in macrophages following liposomal uptake *in vivo*. Interestingly, the effect of non-liposomal tesaglitazar treatments on ATM *Mcp-1* levels was opposite to that of liposomal delivery, suggesting that ATM *Mcp-1* expression was not due to direct PPARα/γ transcription regulation in ATMs. Adipocytes and other stromal cell types, such as ECs and VSMCs, express macrophage function-modulating signals in response to excess lipid loading and hypoxia 69-71 and thus tesaglitazar action in these cell types may results in secretion of molecular signals to induce paracrine effects that regulate ATM populations. Indeed, only about 10% and 40% of CD45^-^ cells in Epid and SC AT, respectively, took up liposomes so drug action in these cell types was likely attenuated relative to non-liposomal tesaglitazar treatments. This may explain why the standard oral formulation of tesaglitazar, but not liposomal delivery was able to induce reduced numbers of ATMs. Development of a targeted liposome construct to deliver tesaglitazar to more CD45^-^ or other non-macrophage subtypes could aid in verifying the hypothesis that tesaglitazar effects in non-macrophage cells influence ATM biology and potentially increase the beneficial metabolic effects of tesaglitazar.

Alternatively, targeting other anti-inflammatory compounds to macrophages in liposomes, using macrophage-specific targeting moieties, or utilizing mechanical manipulation of macrophages via magnetic nanoparticles may also improve metabolic outcomes. Glucocorticoid receptor (GR) agonism has potent anti-inflammatory effects and GR agonists have been used as rheumatoid arthritis therapies, though undesired side effects remain in many patients 72-74. Liposome targeting of a GR agonist to macrophages could be a means of increasing their therapeutic window. Recently, a study utilizing a mannose receptor-targeted nanoparticle to target delivery of PPARα/γ agonist, lobeglitazone, to macrophages in advanced atherosclerotic plaques effectively reduced plaque burden and inflammation 39. Future studies evaluating the capacity of this targeted particle to target ATMs and improve symptoms in an obese, diabetic model may prove successful. Additionally, studies have demonstrated that delivery of magnetic nanoparticles to macrophages followed by induction of varying mechanical forces can alter macrophage phenotype 75, 76. Applying such a technique *in vivo* to induce an anti-inflammatory phenotype may effectively treat symptoms of obesity-associated dysmetabolism.

Finally, we found it surprising that liposomal delivery of tesaglitazar did not change expression of *Mcp-1* in macrophages, given that PPARα and -γ agonists have previously been shown to inhibit MCP-1 expression 33, 77. This led us to question whether PPARα/γ agonism coupled with liposomal uptake differentially affects transcriptional regulation. Indeed, lipids that are commonly used in liposomal formulations have been shown to influence macrophage biology 78, 79. Further investigation is needed to better understand the biological action(s) of these liposomes *in vivo*.

## Conclusions

In summary, this study demonstrates that (1) macrophages are the predominant cell type that takes up drug-loaded liposomes *in vivo,* however our fulsome analysis highlights the need to understand the potential impact of therapeutic drugs in other cell types. (2) For the first time, we demonstrate that a PPARα/γ dual agonist reduces inflammatory macrophages in adipose tissue through paracrine effects. And finally, (3) liposomal delivery is an effective strategy to reduce drug action in hepatocytes and kidneys.

## Supplementary Material

Supplementary figures and tables.

## Abbreviations

AT: adipose tissue
ATM: adipose tissue macrophages
DSPC: 1,2-distearoyl-sn-glycero-3-phosphocholine
DSPE: 1,2-distearoyl-sn-glycero-3-phosphoethanolamine
Ehhadh: enol-CoA hydratase and 3-hydroxyacyl CoA dehydrogenase
EC: endothelial cell
Epid: epididymal
Fabp: fatty acid binding protein
FACS: fluorescently activated cell sorting
FMT: fluorescence molecular tomography
GR: gluococorticoid receptor
Lpl: lipoprotein lipase
MCP-1: monocyte chemoattractant protein-1
MFI: mean fluorescent intensity
Pdk4: Pyruvate dehydrogenase kinase 4
PEG: polyethylene glycol
PerC: peritoneal cavity
PPAR: peroxisome proliferator-activated receptor
RES: reticuloendothelial system
SC: subcutaneous
SD: standard deviation
SVF: stromal vascular fraction
Tbp: tata box binding protein
VSMCs: vascular smooth muscle cells
