# Supplementary material for: In vivo liposomal delivery of PPARα/γ dual agonist tesaglitazar in a model of obesity enriches macrophage targeting and limits liver and kidney drug effects
Source: Theranostics. 2020 Jan 1;10(2):585–601. doi: 10.7150/thno.36572 (PMC6929996; doi:10.7150/thno.36572)
Supplement: Supplementary file 1 — Supplementary figures and tables. [file thnov10p0585s1.pdf]

Table S1: Flow cytometry antibodies

Marker	Fluorochrome(s)	Company	Clone
CD3	FITC	Pharmingen, BD Biosciences	145-2C11
CD11b	BV421, FITC	BioLegend	M1/70
CD11b	PerCP Cy5.5	BD Biosciences	M1/70
CD11c	APC ef780	eBioscience	N418
CD19	PE	eBiosciences	eBio1D3
CD19	PE CF594	BD Biosciences	1D3
CD31	FITC	eBioscience	390
CD31	BV605	Biolegend	390
CD45	PE-CF495, PerCP	BD Biosciences	30-F11
CD115	PE	eBioscience	AFS98
B220	BV421	Biolegend	RA3-6B2
F4/80	PE-Cy7	BioLegend	BM8

Table S2: qRT-PCR primer sequences

Gene	Forward (5’-3’)	Reverse (5’-3’)
18s	CGGCTACCACATCCAAGGAA	AGCTGGAATTACCGCGGC
Arginase-1	AAGACAGCAGAGGAGGTGAAGAG	TGGGAGGAGAACGCGTTTGC
Ehhadh	TCGAATGTTGGCTCCCTATTAC	CCAGCTTCACAGAGCATATCA
Fabp3	AGTCACTGGTGACGCTGGACG	AGGCAGCATGGTGCTGAGCTG
Lpl	GCCGCGTAGTTCCAGCAGCA	CCCTCCTCGGAAGGCGGTCA
Mcp-1	GGTGTCCCAAAGAAGCTGTA	TGTATGTCTGGACCCATTCC
Pdk4	AATTTCCAGGCCAACCAATCC	GGTCAAGGAAGGACGGTTTTC

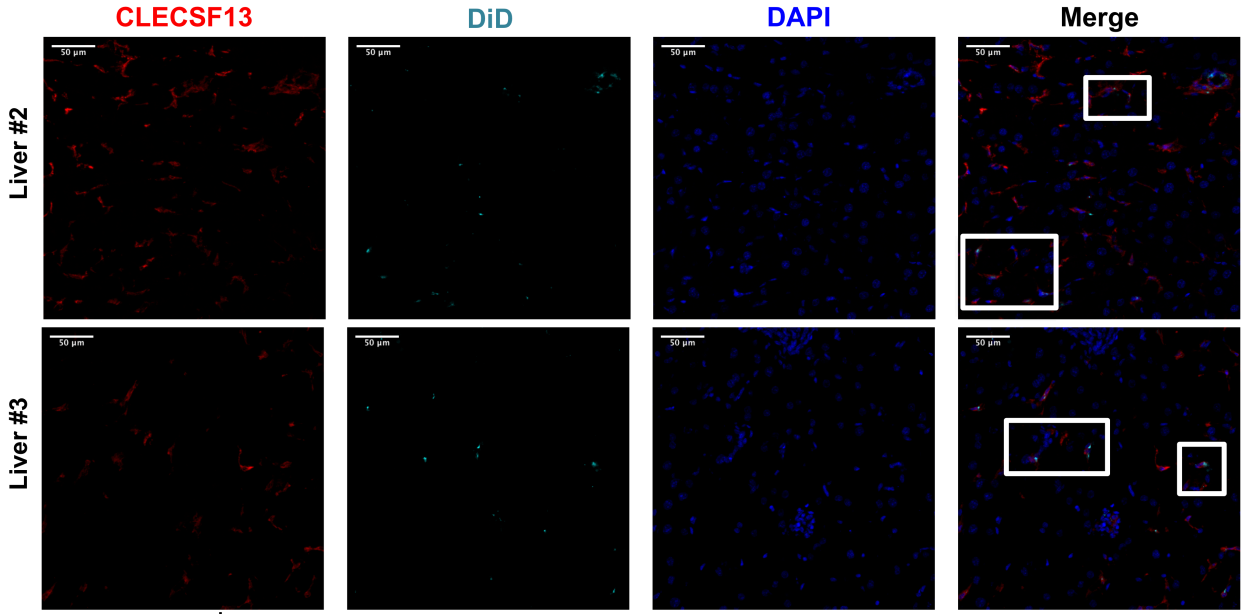

Figure S1: DiD⁺ liposomes co-localized with CLECSF13 in liver. Z-stack images of liver sections from an additional two *ob/ob* mice treated with tesaglitazar-loaded liposomes were stained with CLECS13F to identify Kupffer cells and assessed for co-localization with DiD-labeled liposomes. Co-localization of CLECS13F$^+$ cells and DiD are marked by white boxes.

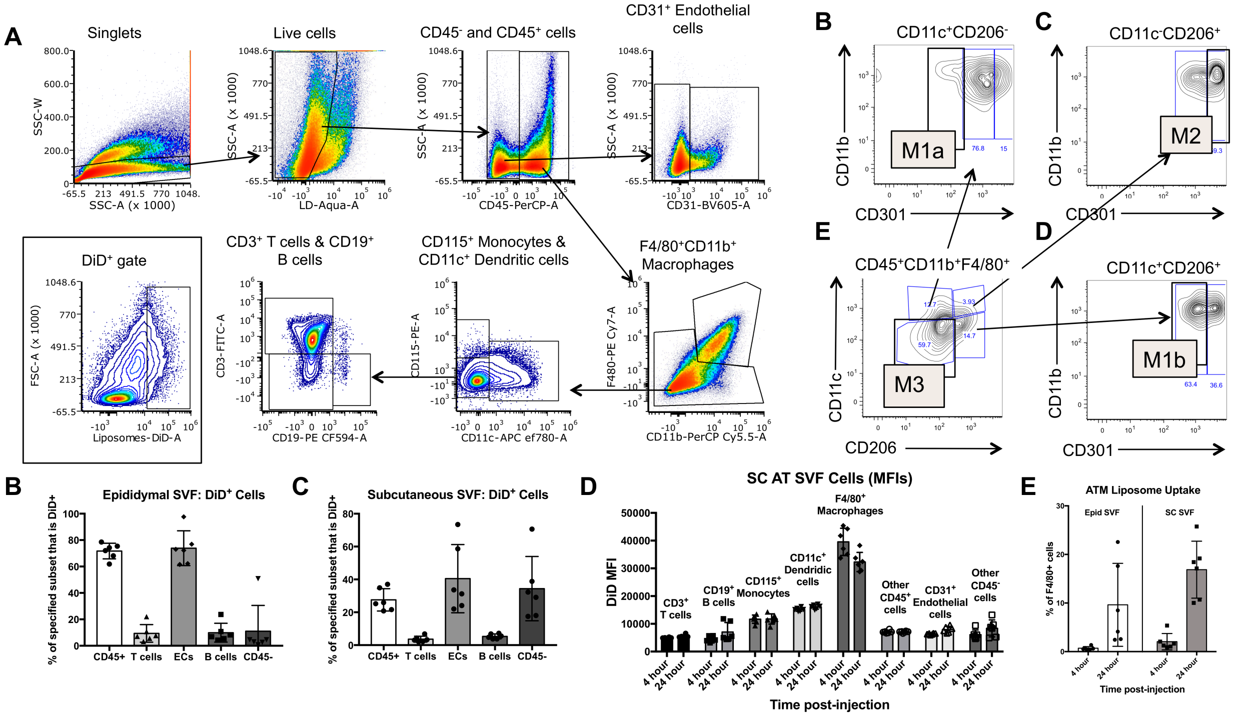

Figure S2: Cell subsets that took up liposomes in the Epid and SC adipose SVF and ATM subset flow cytometry gating strategy. Epid and SC ATs from treated *ob/ob* mice were harvested and processed. Representative flow plots demonstrate flow cytometry gating to identify $CD45^{+}F4/80^{+}$ macrophages, $CD45^{-}CD31^{+}$ endothelial cells, $CD45^{+}CD115^{+}$ monocytes, $CD45^{+}CD11c^{+}$ dendritic cells, $CD45^{+}CD3^{+}$ T cells, $CD45^{+}CD19^{+}$ B cells, and the DiD^{+} population within each of these subsets (A) as well as $CD45^{+}CD11b^{+}F4/80^{+}$ macrophage subsets: $CD11c^{+}CD206^{-}CD301^{-}$ M1a (B), $CD11c^{+}CD206^{+}CD301^{low}$ M1b (E), $CD11c^{-}CD206^{+}CD301^{high}$ M2 (C), and $CD11c^{-}CD206^{-}$ M3 (D). Quantification of the percentage of specified non-macrophage subsets that are DiD^{+} in the Epid SVF (B) and SC SVF (C) after 7 days of liposome treatments. The DiD MFI in all cell subsets of the SC SVF at four- and 24-hour time points were also quantified (D). Finally, the percent of $F4/80^{+}$ macrophages that are DiD^{+} was also quantified in Epid and SC AT at four- and 24-hour time points (E). Mice were treated with tesaglitazar-loaded liposomes. Data represents the mean ± SD

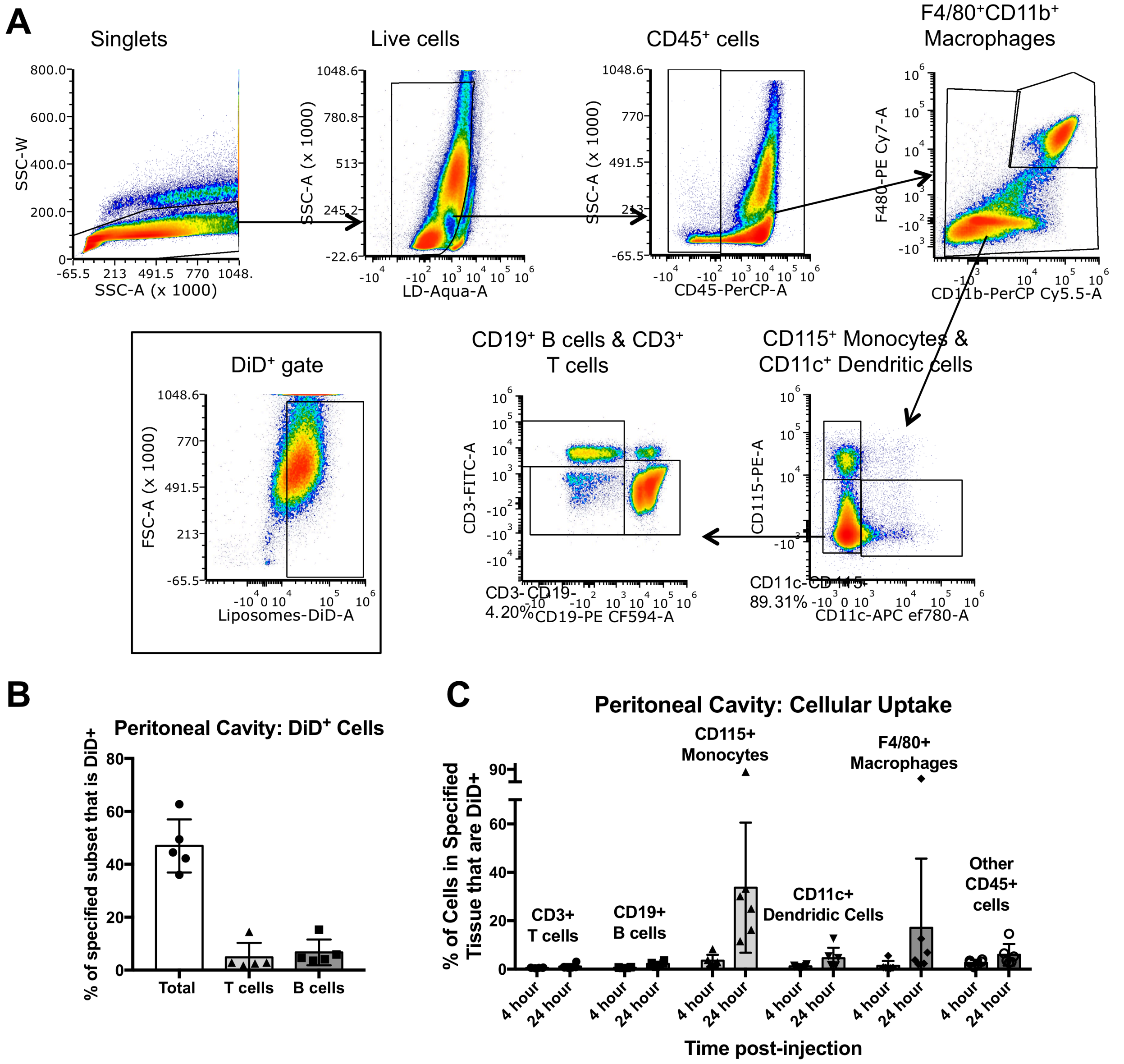

Figure S3: Cell subsets that took up liposomes in the peritoneal cavity and flow cytometry gating strategy. Peritoneal lavages from treated *ob/ob* mice were harvested and processed. Representative flow plots demonstrate flow cytometry gating to identify $CD45^+F4/80^+$ macrophages, $CD45^+CD115^+$ monocytes, $CD45^+CD11c^+$ dendritic cells, $CD45^+CD3^+$ T cells, $CD45^+CD19^+$ B cells, and the DiD^+ population within each of these subsets (A). Quantification of the percentage of non-macrophage cell subsets that are DiD^+ in the peritoneal cavity after seven days of treatment was performed (B). The percent of all subsets that are DiD^+ was also quantified at four- and 24-hour time points (C). Mice were treated with tesaglitazar-loaded liposomes. Data represents the mean ± SD.

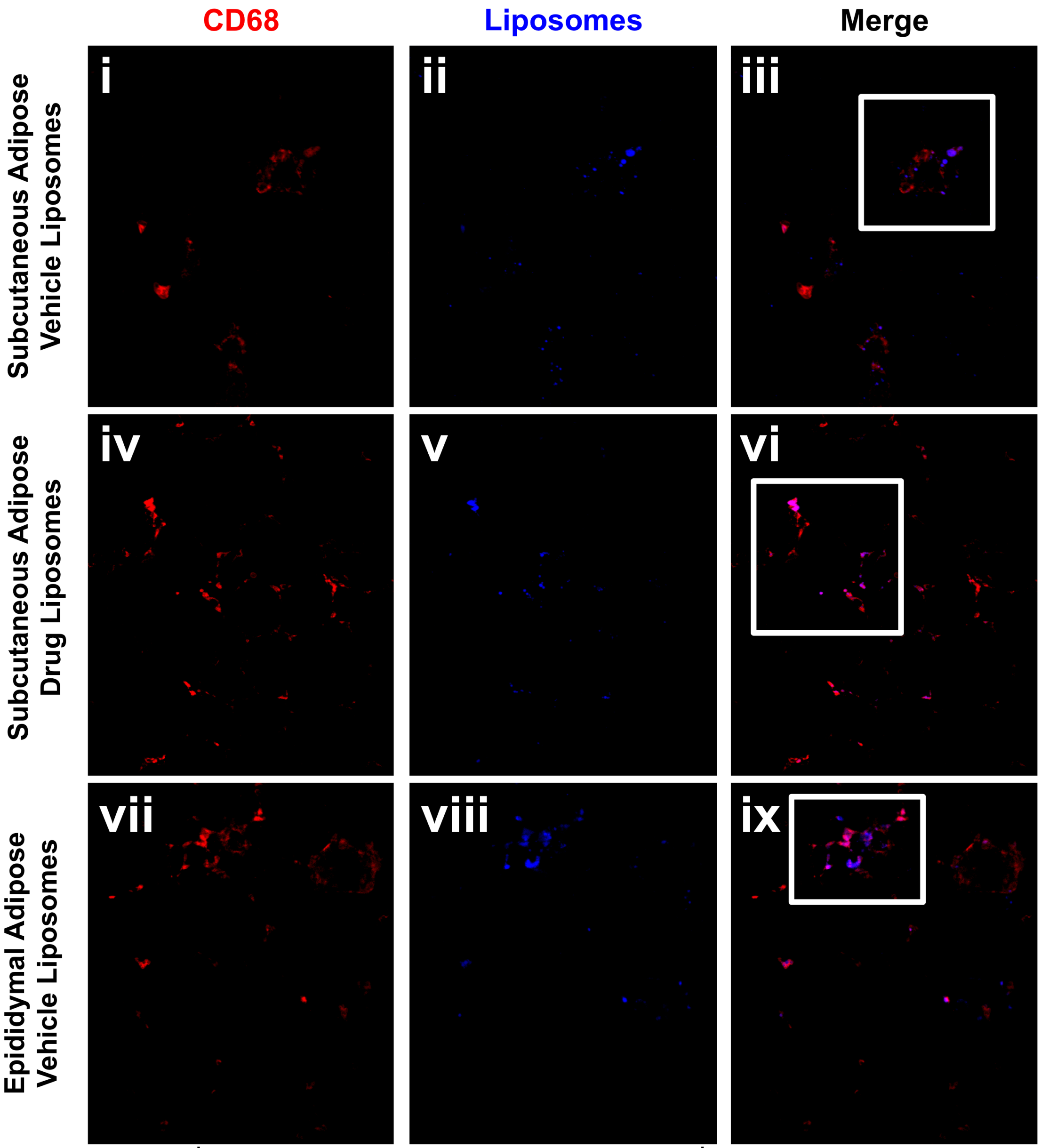

Figure S4: DiD⁺ liposomes co-localized with CD68⁺ macrophages in white adipose tissue and CLECSF13 in liver. 40-μm Z stack images of whole mounted SC AT (Ai-vi) or Epid AT (Avii-ix) from *ob/ob* mice treated with vehicle- (Ai-iii, Avii-ix) or drug-loaded (Aiv-vi) liposomes were stained with CD68 to mark macrophages and assessed for co-localization with DiD-labeled liposomes. Co-localization of interstitial CD68⁺ cells and DiD are marked by white boxes.

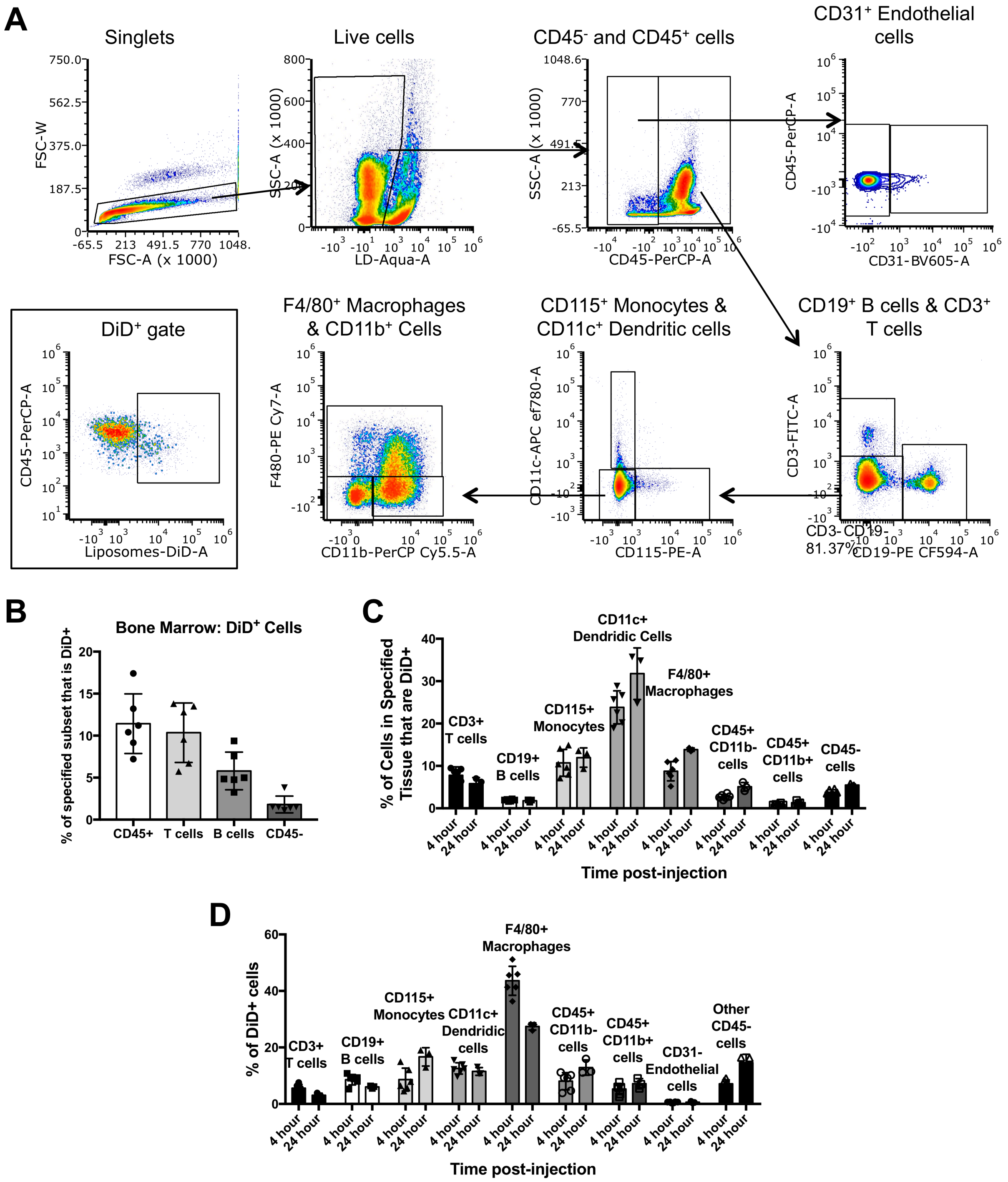

Figure S5: Cell subsets that took up liposomes in the bone marrow and flow cytometry gating strategy. Representative flow plots demonstrate flow cytometry gating to identify $CD45^{+}F4/80^{+}$ macrophages, $CD45^{-}CD31^{+}$ endothelial cells, $CD45^{+}CD115^{+}$ monocytes, $CD45^{+}CD11c^{+}$ dendritic cells, $CD45^{+}CD3^{+}$ T cells, $CD45^{+}CD19^{+}$ B cells, other $CD45^{+}CD11b^{+}$ cells that include cells such as NK cells and neutrophils, and the DiD^{+} population within each of these subsets (A). Quantification of the percentage of cells subsets that are DiD^{+} in the peritoneal cavity after seven days (B) or four- and 24-hour time points (C) was performed. The proportion of DiD^{+} cells that are represented by each cell subset was also calculated (D). Mice were treated with tesaglitazar-loaded liposomes. Data represents the mean ± SD.

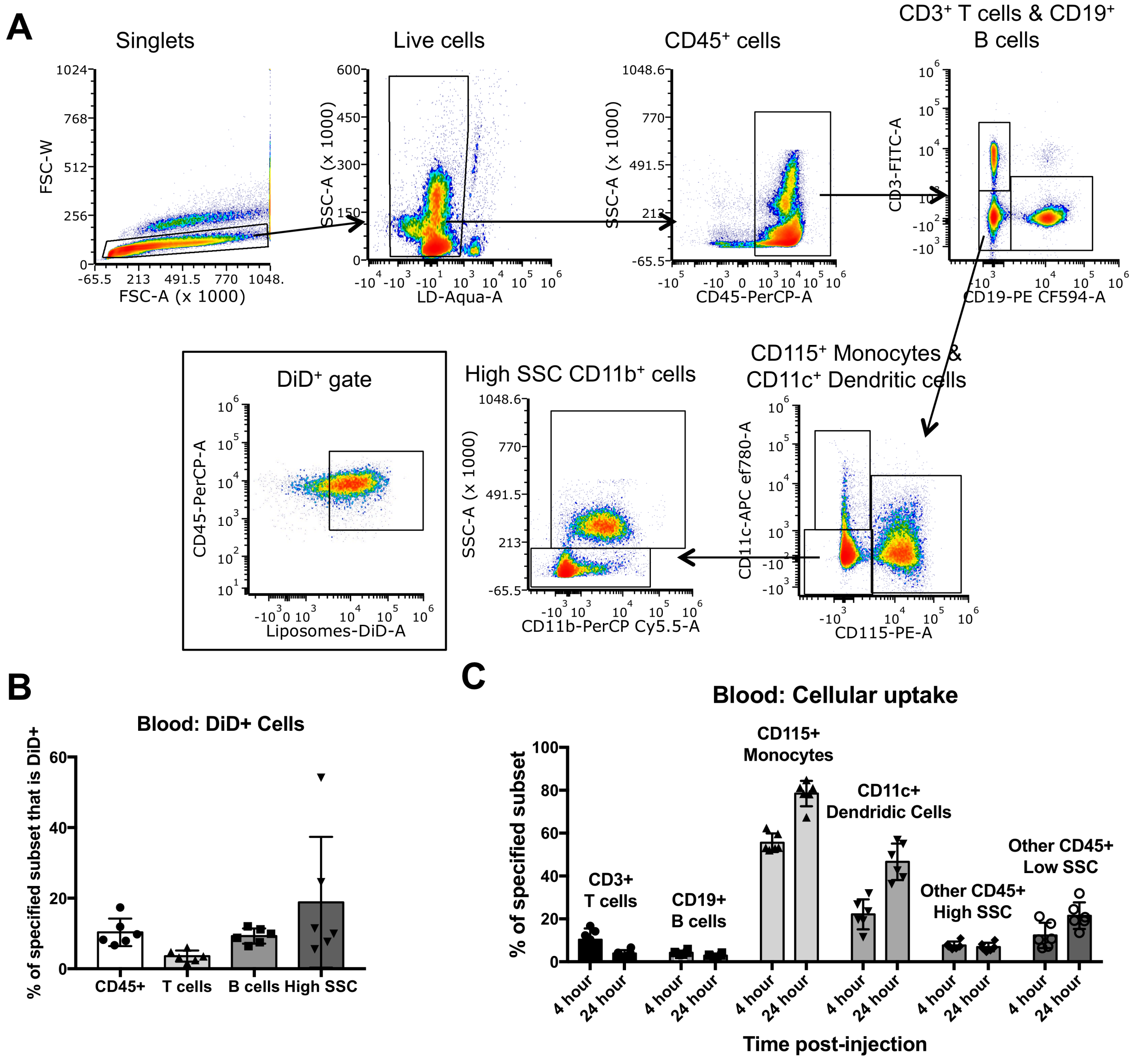

Figure S6: Cell subsets that took up liposomes in the blood and flow cytometry gating strategy. Representative flow plots demonstrate flow cytometry gating to identify $CD45^+CD115^+$ monocytes, $CD45^+CD11c^+$ dendritic cells, $CD45^+CD3^+$ T cells, $CD45^+CD19^+$ B cells, other $CD45^+CD11b^+SSC^{high}$ cells that include neutrophils, $CD45^+$ $CD115^-CD11c^-CD3^-CD19^-SSC^{low}$ that include NK cells, and the DiD^+ population within each of these subsets (A). Quantification of the percentage of specified subsets that are DiD^+ in the blood after seven days (B) or four- and 24-hour time points (C) was performed. Mice were treated with tesaglitazar-loaded liposomes. Data represents the mean ± SD.

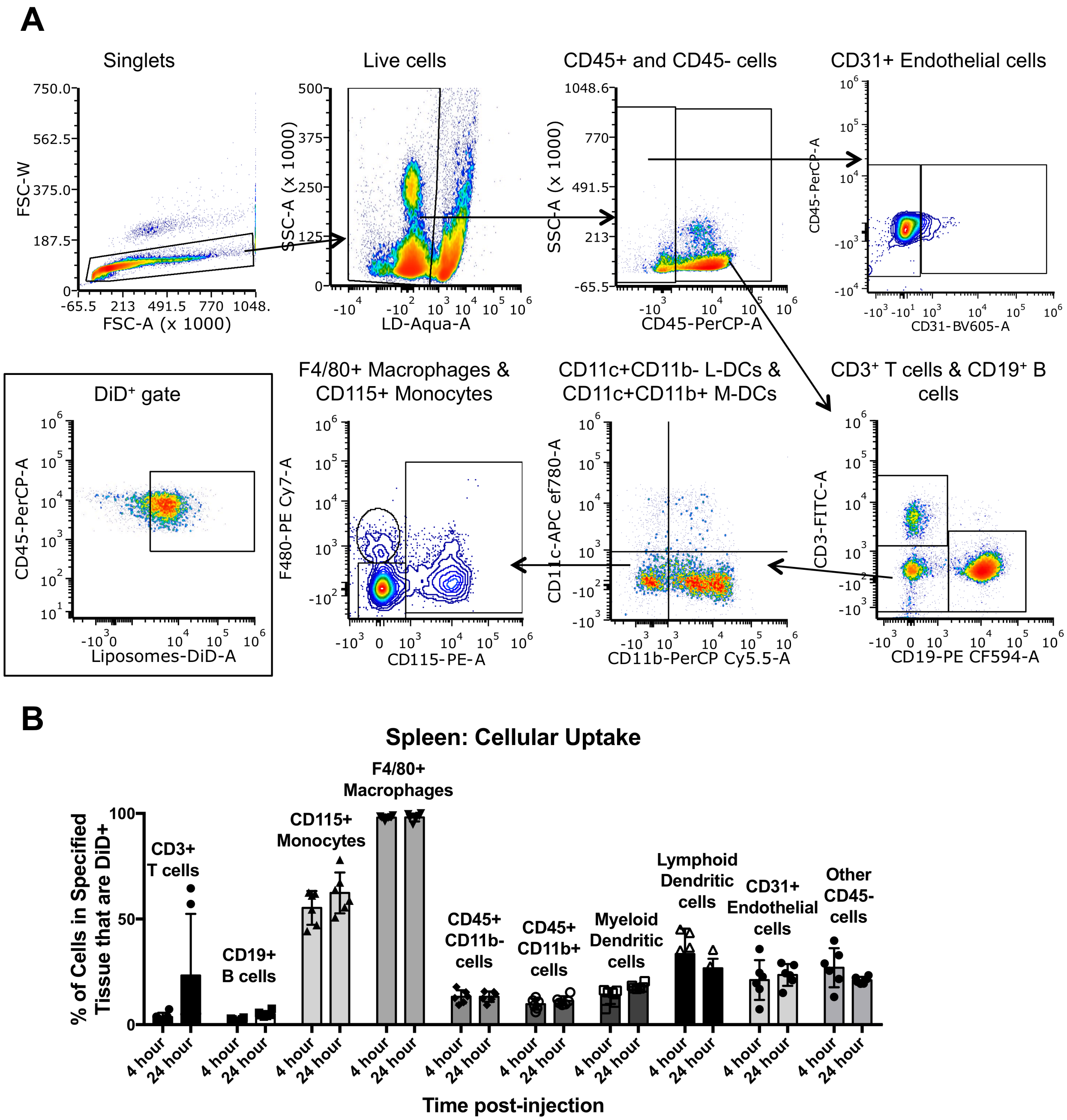

Figure S7: Cell subsets that took up liposomes in the spleen and flow gating strategy. Representative flow plots demonstrate flow cytometry gating to identify CD45$^+$F4/80$^+$ macrophages, CD45$^-$CD31$^+$ endothelial cells, CD45$^+$CD115$^+$ monocytes, CD45$^+$CD11c$^+$CD11b$^-$ lymphoid dendritic cells, CD45$^+$CD11c$^+$CD11b$^+$ myeloid dendritic cells, CD45$^+$CD3$^+$ T cells, CD45$^+$CD19$^+$ B cells, and the DiD$^+$ population within each of these subsets (A). Quantification of the percentage of specified subsets that are DiD$^+$ in the blood at four- and 24-hour time points (B) was performed. Mice were treated with tesaglitazar-loaded liposomes. Data represents the mean ± SD.

	Marker	$CD45^+CD3^+$	$CD45^+CD19^+$	$CD45^+CD115^+$	$CD45^+CD11c^+$	$CD45^+F4/80^+$	$CD45^+CD3^-CD19^-$	$CD45^-CD31^-$	$CD45^-CD31^+$
	**Cell type**	T Cells	B Cells	Monocytes	Dendritic Cells	Macrophages	Other $CD45^+$ Cells	Other $CD45^-$ Cells	Endothelial Cells
**Time post-injection**	**Epididymal Adipose SVF**								
4h	Average	4.74	0.04	0.75	4.55	6.54	13.00	34.75	35.62
	Standard Dev	0.65	0.03	0.23	0.67	2.00	0.80	6.37	5.59
24h	Average	6.17	0.03	0.50	8.24	25.91	24.39	12.60	22.15
	Standard Dev	0.78	0.02	0.17	2.54	9.70	4.93	2.41	5.48
	**Subcutaneous Adipose SVF**								
4h	Average	10.33	0.26	1.25	4.37	5.38	23.58	26.23	28.58
	Standard Dev	0.68	0.53	0.15	1.64	4.06	1.15	5.55	3.89
24h	Average	11.69	0.49	0.90	4.65	20.13	31.95	13.02	17.17
	Standard Dev	1.02	0.99	0.22	1.40	6.15	3.91	3.57	3.79

Figure S8: Proportion of cell subsets that were DiD+ in adipose SVF at early time points. Table reporting the percent of DiD^+ cells in the Epid and SC SVF that are $CD45^+F4/80^+$ macrophages, $CD45^-CD31^+$ endothelial cells, $CD45^+CD115^+$ monocytes, $CD45^+CD11c^+$ dendritic cells, $CD45^+CD3^+$ T cells, $CD45^+CD19^+$ B cells, and other $CD45^+$ or $CD45^-$ cells. Uptake percentages are reported at four- and 24 hours post-injection. These data provide quantification for the pie charts reported in Figure 3A-D, n = 6 per time point.

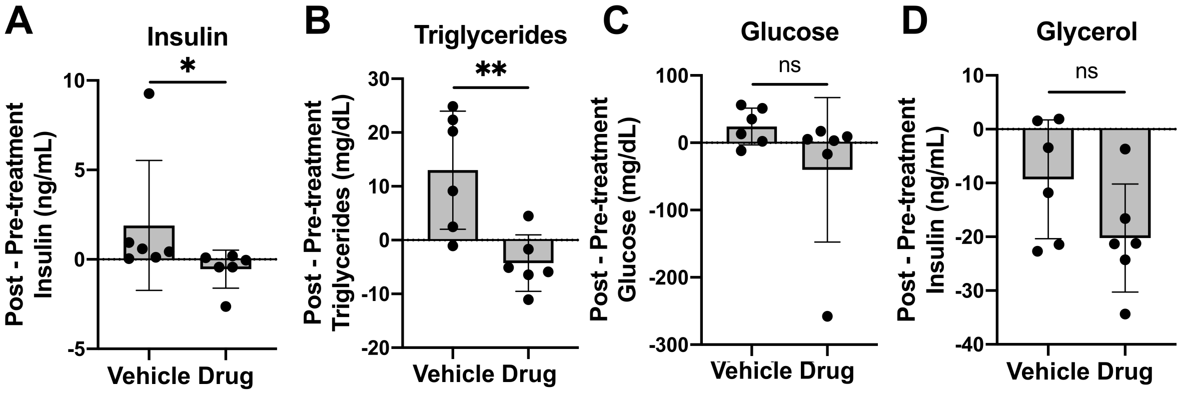

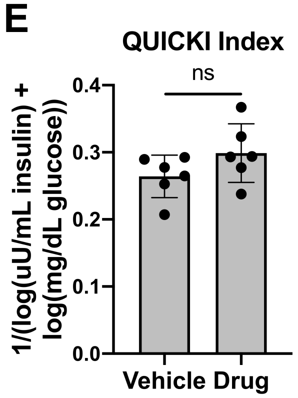

F

Gene expression fold change

		ob/ob$_{Drug}$ / *ob/ob*$_{Vehicle}$	DIO_{Drug} / $DIO_{Vehicle}$	*ob/ob*$_{Drug}$ / DIO_{Drug}
**Liver**	*Ehhadh*	36.55	17.75	2.06
	Lpl	28.01	18.30	1.53
	Fabp3	447.24	292.11	1.53
**Epididymal**	*Ehhadh*	37.82	12.46	3.03
	Adiponectin	1.93	0.78	2.49
	Lpl	7102.18	4708.94	1.51

Figure S9: Effects of tesagltizar treatments on metabolic parameters and gene expression in a high-fat diet-induced obesity model. Male DIO mice were treated daily by oral administration of tesaglitazar or vehicle for seven days. To assess metabolic effects, blood was harvested from mice before and after treatments and plasma was harvested. Circulating insulin (A), triglycerides (B), glucose (C), and glycerol (D) levels before and after treatment were measured and the changes in levels per mouse were calculated. Post-treatment glucose and insulin levels were also utilized to quantify QUICK index for each mouse (E). RNA extracted from livers and epidydmal adipocytes of each DIO and *ob/ob* mice treated for seven days with vehicle or tesaglitazar (as free drug) were used to quantify relative gene expression of *Ehhadh*, *Lpl*, and *Fabp3* in the liver and *Ehhadh*, *Adiponectin*, and *Lpl* in epididymal adipocytes by qRT-PCR. Fold change between ob/ob mice treated with drug (*ob/ob*$_{Drug}$) and ob/ob mice treated with vehicle (*ob/ob*$_{Vehicle}$) were calculated, as were DIO treated with drug (DIO_{Drug}) compared to DIO treated with vehicle ($DIO_{Vehicle}$). To compare gene expression levels in each drug-treated group, *ob/ob*$_{Drug}$ expression values were divided by DIO_{Drug} values (F). Data represents the mean ± SD; * $p \leq 0.05$, ** $p \leq 0.01$. Vehicle indicates animals orally administered vehicle, drug indicates animals orally administered tesaglitazar.

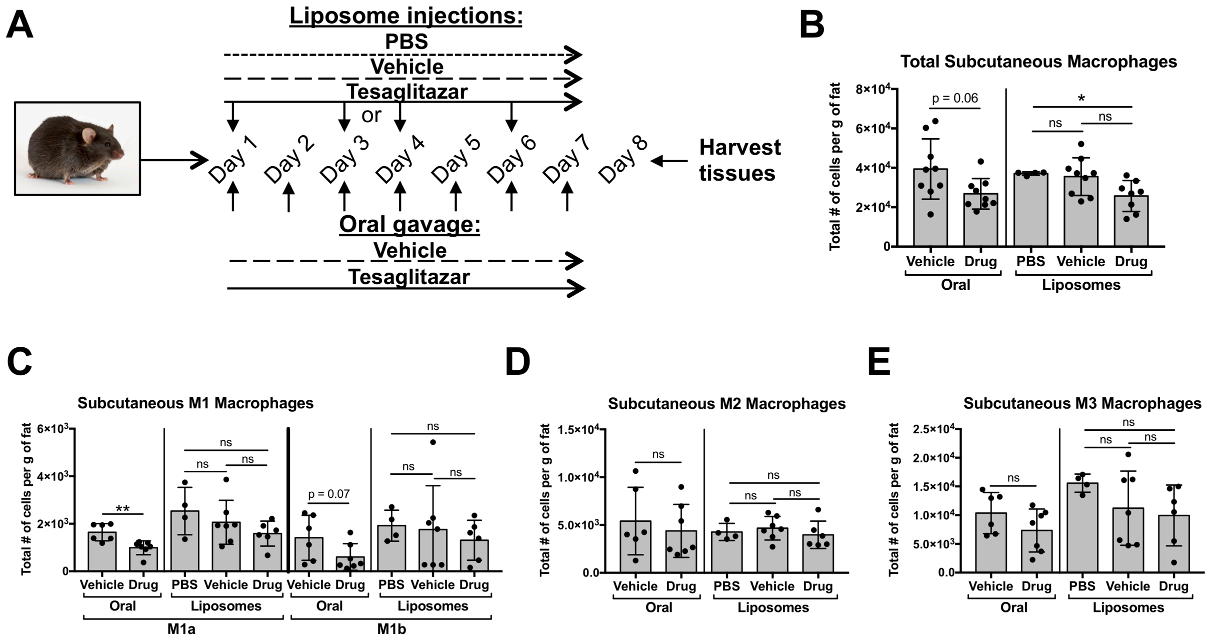

Figure S10: Effects of free drug and liposomal delivery of tesaglitazar on subcutaneous adipose tissue macrophage populations. SC SVF cells from *ob/ob* mice treated for seven days via oral administration or liposomal delivery were stained with antibodies against markers of macrophages and macrophage subsets to quantify cell numbers by flow cytometry (A). Total $CD45^{+}CD11b^{+}F4/80^{+}$ macrophage numbers from SC adipose were normalized to the total mass of the adipose depot (B). M1a and M1b (C), M2 (D), and M3 (E) macrophage subsets were quantified and normalized to total adipose mass as well. Data represents the mean ± SD; * $p \leq 0.05$, ** $p \leq 0.01$.
